# Evidence for Diversity in Transcriptional Profiles of Single Hematopoietic Stem Cells

**DOI:** 10.1371/journal.pgen.0020159

**Published:** 2006-09-29

**Authors:** Carlos A Ramos, Teresa A Bowman, Nathan C Boles, Akil A Merchant, Yayun Zheng, Irma Parra, Suzanne A. W Fuqua, Chad A Shaw, Margaret A Goodell

**Affiliations:** 1 Department of Medicine, Baylor College of Medicine, Houston, Texas, United States of America; 2 Center for Cell and Gene Therapy, Baylor College of Medicine, Houston, Texas, United States of America; 3 Cell and Molecular Biology Program, Baylor College of Medicine, Houston, Texas, United States of America; 4 Breast Center, Baylor College of Medicine, Houston, Texas, United States of America; 5 Human and Molecular Genetics, Baylor College of Medicine, Houston, Texas, United States of America; 6 Department of Pediatrics, Baylor College of Medicine, Houston, Texas, United States of America; Stanford University School of Medicine, United States of America

## Abstract

Hematopoietic stem cells replenish all the cells of the blood throughout the lifetime of an animal. Although thousands of stem cells reside in the bone marrow, only a few contribute to blood production at any given time. Nothing is known about the differences between individual stem cells that dictate their particular state of activation readiness. To examine such differences between individual stem cells, we determined the global gene expression profile of 12 single stem cells using microarrays. We showed that at least half of the genetic expression variability between 12 single cells profiled was due to biological variation in 44% of the genes analyzed. We also identified specific genes with high biological variance that are candidates for influencing the state of readiness of individual hematopoietic stem cells, and confirmed the variability of a subset of these genes using single-cell real-time PCR. Because apparent variation of some genes is likely due to technical factors, we estimated the degree of biological versus technical variation for each gene using identical RNA samples containing an RNA amount equivalent to that of single cells. This enabled us to identify a large cohort of genes with low technical variability whose expression can be reliably measured on the arrays at the single-cell level. These data have established that gene expression of individual stem cells varies widely, despite extremely high phenotypic homogeneity. Some of this variation is in key regulators of stem cell activity, which could account for the differential responses of particular stem cells to exogenous stimuli. The capacity to accurately interrogate individual cells for global gene expression will facilitate a systems approach to biological processes at a single-cell level.

## Synopsis

The hematopoietic stem cell (HSC) has the remarkable property of being able to generate more stem cells or cells that are committed to undergo differentiation into specific blood lineages. Currently, very little is known about the specific mechanisms that underlie self-renewal or lineage commitment. Although it is possible that some of these mechanisms are influenced by the specific environment in which the HSC dwells, the ultimate fate decision has to occur at the single HSC level. The authors have developed a method that amplifies the messages from the majority of genes that are active in a single stem cell and combines it with large-scale genetic expression analysis through the use of nucleic acid microarrays. A significant fraction of these genes are found to be highly variable in an apparently very homogeneous stem cell population, which could be the substrate for differences in behavior of individual stem cells. Understanding the genetic expression events at the single-cell level would grant the ability to expand HSCs or to direct their differentiation into specific populations, both important from a therapeutic point of view. Furthermore, the same techniques can be applied to other stem cell systems to investigate their physiology.

## Introduction

Interest in adult stem cells has intensified since 2002, due to renewed hope for their application to regenerative medicine [1–8]. The adult hematopoietic stem cell (HSC), a paradigm for understanding the mechanisms that regulate stem cell generation and regulation, resides primarily in a quiescent state in the bone marrow until recruited to generate differentiated blood cells. Although an adult mouse harbors hundreds of HSCs, only between one and ten are thought to be active in contributing to blood production at any time [9,10]. Nothing is known about the mechanisms that favor activation of one stem cell over another. Presumably, apart from micro-environmental factors, there are individual differences in the ability of particular stem cells to respond, based on a constellation of response genes they express at a given time. Although several efforts have been made to study the transcriptional profile of HSC at the population level [11–17], the ability to investigate gene expression in stem (and other) cells at the single-cell level would be a powerful tool to understand their biology.

In addition, the most restrictively defined HSC populations have not always been proven to be 100% functionally homogeneous with regard to both differentiation and self-renewal [18]. Although part of this inefficiency could be explained by technical limitations, the successive description of new surface markers to further enrich stem cell populations that were previously thought to be “pure” [19–21] seems to demonstrate that those are not the only factors to blame. Furthermore, there is good evidence that highly purified HSC populations, such as the side population (SP), can be fractionated into sub-populations that possess distinct potential [22]. All these observations strongly argue that stem cell populations defined by current methods are heterogeneous. Studying this heterogeneity will offer important insights regarding stem cell physiology. Again, given the minimal number of cells that compose highly enriched populations, and their subsets, one needs to be able to study global genetic expression starting with minute numbers of cells.

A few studies have addressed single cell transcriptional profiling, but the methods have been applied to study a limited number of genes [23–25] or have not been entirely validated [26]. We have developed a strategy to amplify transcripts from small numbers of cells that combines a method to amplify mRNA from single cells (global single-cell RT-PCR or GSC RT-PCR), initially described by Brady and collaborators [27,28], and high-throughput genomic technology, using Affymetrix microarrays [29]. Using this strategy, we were able to accurately represent the transcriptional profiles of minimal numbers of cells, including single cells. This has allowed us to demonstrate the presence of micro-heterogeneity in gene expression among cells of an otherwise phenotypically homogenous stem cell population.

## Results

### GSC RT-PCR Combined with Oligonucleotide Microarray Analysis

In our experiments, murine bone marrow HSCs were purified on the basis of their differential Hoechst dye efflux ability (SP cells) [30], as well as their expression of the stem cell antigen Sca-1 [19], and lack of the myeloid lineage marker Gr-1. Our group has recently demonstrated that SP cells are phenotypically Lin^−/low^Sca-1^+^c-kit^+^Thy1.1^low^ CD34^neg/low^Flk2^neg^ and thus are, in essence, the same HSC population isolated by other groups. Around 67% and 74% of wells sorted with single SP or Lin^−/low^Sca-1^+^c-kit^+^CD34^neg/low^ (KLS34) cells, respectively, contained large multi-lineage colonies [31]. When we sorted KLS34 cells that had also been stained with Hoechst, around 65% of the wells were clonogenic, indicating that Hoechst dye is only slightly toxic to HSC. Under optimal conditions, single cells close to the SP tip are able to engraft long-term in approximately one third (35%) of lethally irradiated recipients, frequencies that compare favorably with the 20% to 33% obtained by other authors, all of which used purification strategies based on surface antigen expression [18,21,32]. Therefore, the HSC population we used is highly homogeneous at a phenotypic and functional level [22]. We will refer to the population hereafter as simply SP cells.

One or ten SP cells were lysed in a first strand buffer and a limited reverse transcription was performed, generating limited-size cDNAs that represent the 3′-most few-hundred nucleotides of most mRNA molecules in solution. These products were then polyadenylated and amplified in a PCR reaction primed by an oligonucleotide containing a poly-T tract (Figure S1). The limited reverse transcription ensures that all the first strands have approximately the same size, which reduces bias in the PCR amplification potentially caused by differences in amplicon sizes [28]. Because the material generated in this process is dsDNA and not biotinylated aRNA, we had to adapt the conventional Affymetrix protocol by end-labeling the cDNA with biotinylated ddATP [33,34], after determining the optimal conditions for its random digestion with DNase I into fragments averaging 50 bp. The targets thus produced were then incubated with Murine Genome U74A version 2 microarrays according to the standard protocol (Affymetrix, Santa Clara, California, United States). Because the probes present on these arrays correspond mostly to the 3′ ends of the genes represented, the fact that these targets are 3′ biased should not affect the results significantly, provided that an appropriate method of analysis is chosen. Specific transcript abundance was calculated using an implementation of the RMA procedure. For simplicity, we will use the term “gene expression level” to mean transcript abundance of a particular gene, even though we are not exactly looking at transcriptional rates.

### Amplification and Analysis from Ten-Cell Samples

To validate the strategy with a minimum of biological variation introduced by individual cells being in diverse states of activity (such as different cell cycle stage), we initially amplified transcripts from ten cells and pooled together four of these 10-cell derived amplifications. This strategy enabled us to run replicates of the same amplified samples and show that the results obtained with replicates are reproducible (Figure S2).

Even though a modification of the GSC RT-PCR had been shown to preserve transcriptional representation faithfully when applied to sub-picogram quantities of purified mRNA (diluted from a sample initially isolated from a large number of cells) [35], our overall experimental approach had not been tested previously. Consequently, it needed to be validated for accuracy of relative gene expression level determination and for power to discriminate between samples of different cell types. In order to do this, we compared the genetic expression profile of SP cells to another small, well-defined bone marrow population, CD8 positive T lymphocytes. The expression levels from six independent SP and three CD8 samples run in duplicate were determined using the RMA algorithm [36,37].

### Power of Discrimination

**Figure 1:**
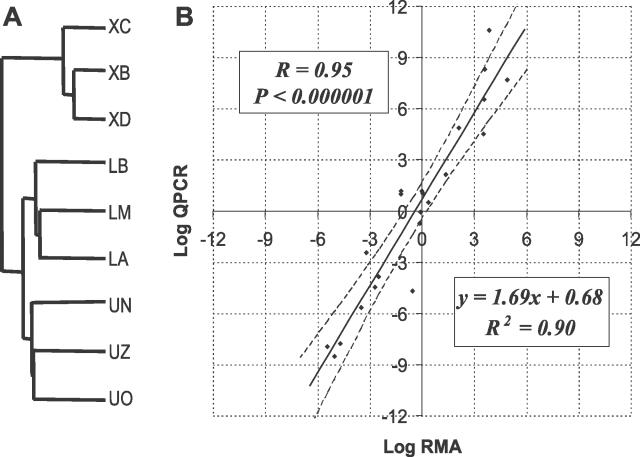
Expression Levels Determined by RMA Using GSC RT-PCR Discriminate between Different Cell Populations and Correlate Tightly with Those Determined by Q-PCR (A) The figure represents a dendrogram for genes consistently above background level in more than 50% of the samples. All the genes were used, i.e., no prior selection for genes differentially expressed in the different populations was done. Each 10-cell sample of the lower region of SP cells (LA, LB, and LM), the upper region of SP cells (UN, UO, and UZ), or CD8 T lymphocytes (XB, XC, and XD) with two replicates averaged is represented. Unsupervised hierarchical clustering based on the Euclidean distance between expression levels for each gene separates clearly the three groups of samples. (B) Twenty-two genes representing the full range of fold changes were selected for analysis. Relative expression levels (fold changes) obtained by RMA performed on microarrays prepared with our amplification procedure (horizontal axis) were plotted against those calculated using real-time Q-PCR (vertical axis). The regression equation and correlation coefficient are shown in the graph. The 95% confidence intervals for the regression are represented by the dashed lines.

Non-supervised hierarchical clustering of samples based on the expression levels of genes above background allowed discrimination between all groups of samples (Figure 1A). In other words, our amplification and detection methods yield genetic expression data that can be used to identify biologically distinct cell populations. Statistical comparison of the average expression level for each gene incorporating variance information through t-statistics defined three groups of genes: predominant in SP (1,315 genes), predominant in CD8 (1,319 genes), and not statistically different (see Table 1 for selected genes and Table S1; all primary data from this study are summarized in Tables S5 and S6, and are also available for download from http://www.ncbi.nlm.nih.gov/geo, series GSE2534). The criterion used to establish a difference was a Student's t-test statistic with an associated single gene *p*-value < 0.05.

**Table 1:**
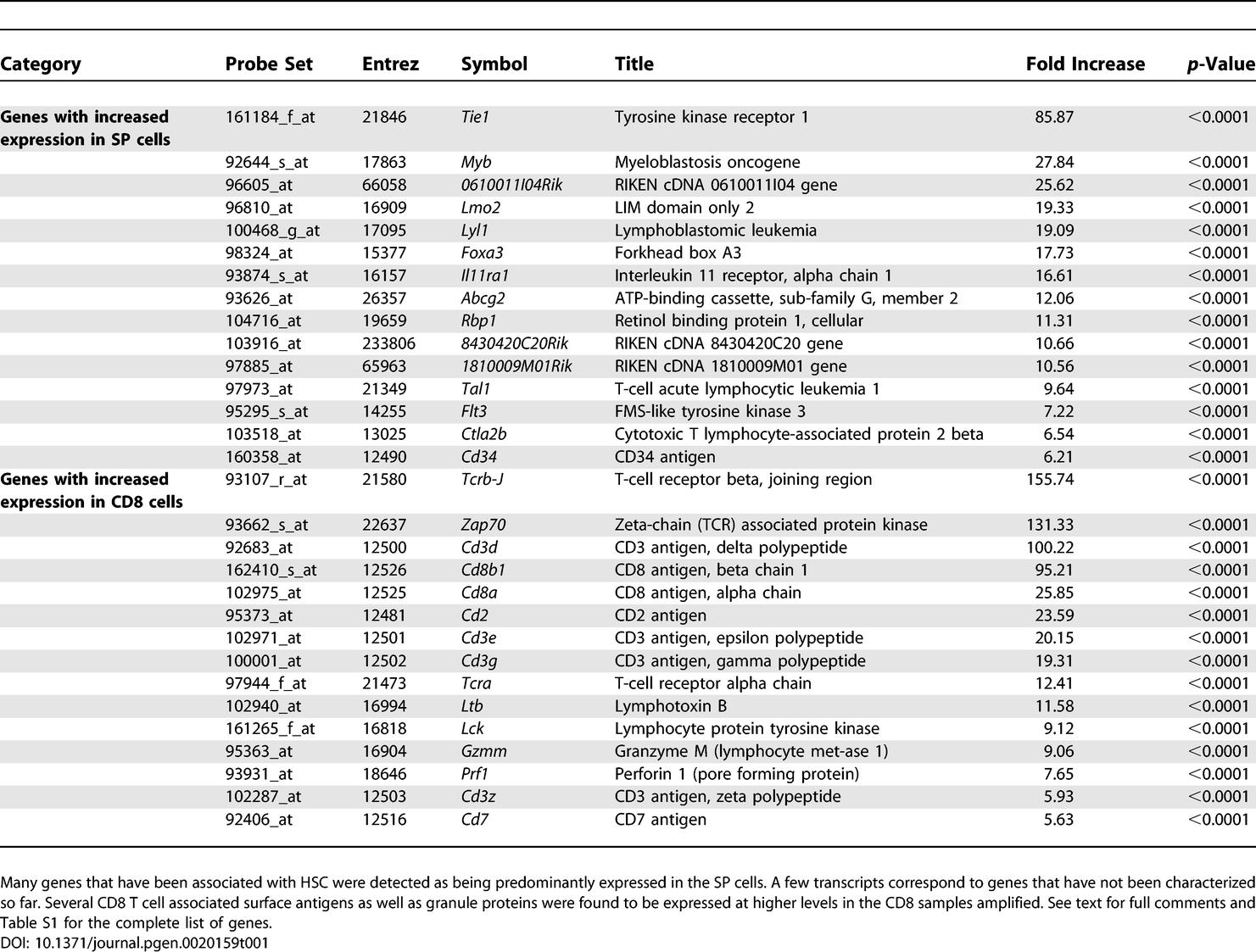
Selected Genes Found to be Differentially Expressed between SP Stem Cells and CD8 T Lymphocytes Based on the Amplification of 10 Cells

### Accurate Prediction of Differences in Transcript Abundance

To validate the expression levels obtained by our amplification strategy using real-time quantitative PCR (Q-PCR), we selected 22 genes representing the full range of fold changes, and designed PCR primer pairs for them. Relative expression levels were then calculated using Q-PCR performed in duplicate on two different samples of RNA. The un-amplified RNA samples were isolated from pools of 200,000 SP cells or 900,000 CD8 cells and an amount of RNA corresponding to approximately 2,500 cells was used in each reaction. The fold changes computed based on RMA data were plotted against those obtained from Q-PCR experiments (Figure 1B and Table S2). We observed a 95% correlation coefficient (*p* < 0.000001) between both sources of data. This establishes that this PCR-based amplification method, coupled with microarray analysis, accurately measures expression levels to a remarkable degree. Consistent with that reported by others [38], the fold changes determined by microarray analysis underestimate the true results calculated from Q-PCR experiments, as can be appreciated by the fact that the slope of the regression line is greater than 1.

### Biological Relevance of Gene Expression Differences

In addition to being quantitatively accurate, the data collected exhibit biological relevance. Comparison of stem cells from a largely quiescent population with cells from an equally resting population should emphasize cell type-specific differences in gene expression and disregard inactive genes or unspecific transcripts, such as those derived from housekeeping genes. Indeed, several genes that are expected to be preferentially expressed in CD8 cells, such as T-cell receptor complex proteins (CD3 subunits and zeta chain, and associated kinases) and the co-receptor CD8 chains among others, show significantly higher expression when compared to SP cells. Conversely, genes known to be associated with the SP and HSC, including *Scl/Tal-1, Ctla-2* [12,14], *Mdr* and *Abcg2* [39], and others, have higher levels of expression in SP cells (Table 1 and Table S1). Therefore, our strategy is reliable for identifying genes that are associated with particular populations.

### Heterogeneity in SP Cells

We then applied these methods to investigate the degree of heterogeneity in stem cell populations, comparing cells isolated from different regions of the SP. Functional studies have shown that cells appearing closer to the tip of the SP (lower SP: LSP), thus displaying higher dye efflux capacity, possess more long term hematopoietic reconstitution capacity than those closer to its shoulder (upper SP: USP) [22]. Notably, phenotypic analysis of surface membrane determinants in these cells shows no major differences between these subpopulations. Microarray analysis reveals that these subpopulations are much more similar to each other than the SP cells are to CD8 T cells, with only 1,082 genes being differentially expressed. Some of these genes are listed in Table 2, with the full table in Table S3. Analysis of the biological function of the products of these genes shows important findings. Several of the predominant transcripts in the LSP correspond to poorly characterized transcription factors. Examples include Runt related transcription factor, Ring finger protein 1, Twisted gastrulation protein, and X-linked Zinc finger protein. In contrast, USP cells seem to express transcripts present in activated cells, such as E2F transcription factor 1, Cyclin A2, Signal-induced proliferation associated gene 1, and products associated with differentiating hematopoietic lineages, such as hemoglobin subunits, CD14 (Myeloid Differentiation Antigen) and Ly6C. Of note, these cells are CD45 positive, i.e. are already committed to a hematopoietic fate, but they are still negative for surface membrane lineage markers, such as those present in erythrocytic and granulocytic lineages. Thus, it appears that these cells may be already turning on genes related to lineage determination, while still not displaying lineage markers.

**Table 2:**
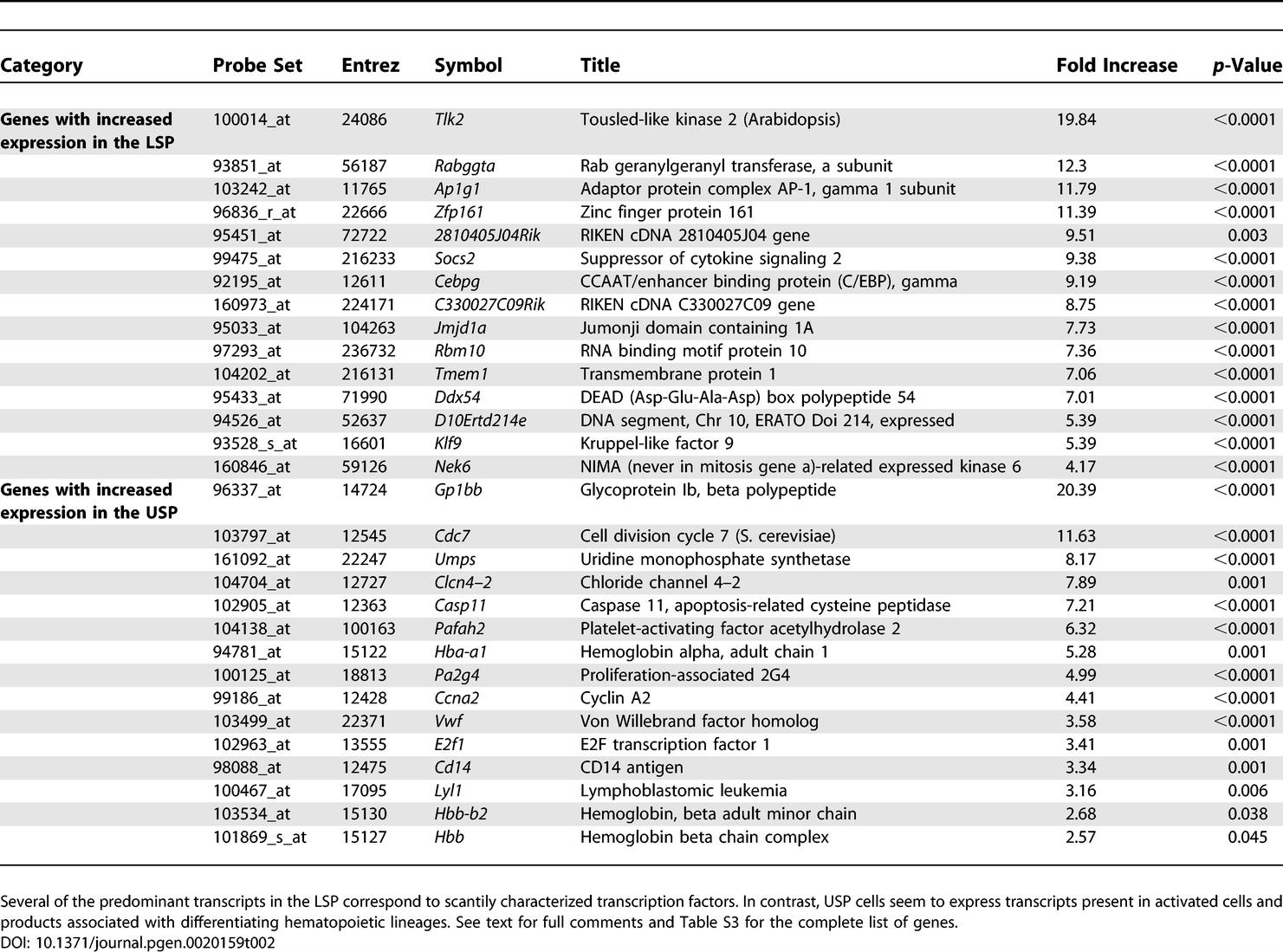
Selected Genes Found to be Differentially Expressed between the Lower and the Upper SP Cells Based on the Amplification of Ten-Cell Samples

### Gene Expression Profile of Single Stem Cells

**Figure 2:**
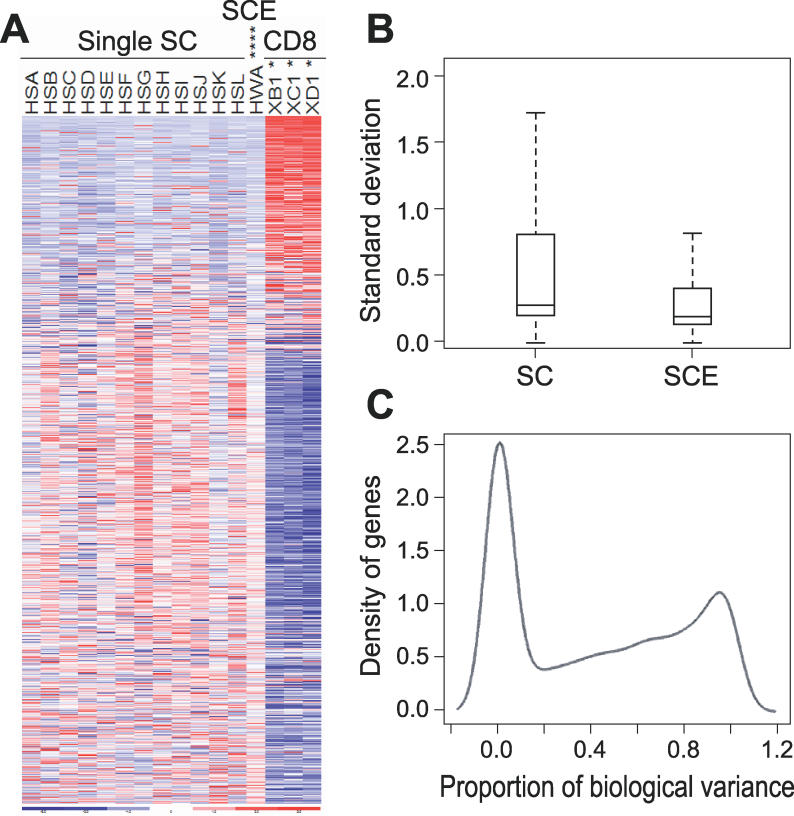
Genetic Representation of Single-Cell Amplifications (A) The transcriptional profiles obtained for individual SP stem cells maintain an accurate representation of the overall genetic differences between populations. The genes that are statistically most different between SP cells and CD8 T lymphocytes (*p* < 0.005), as determined by the 10-cell amplification experiments, are displayed in the heat map. Expression levels were normalized to 1 (white). Red genes are over-expressed and blue genes under-expressed in that sample. HSA through HSL are the results of the amplification of individual single SP cells mRNA. HWA is an average obtained for SCE amplifications (see text for explanation). XB1 through XD1 are the results for 10 CD8 T-cell amplifications. Genes that are expressed at higher levels in CD8 cells are generally expressed at lower levels in the individual SP cells and vice versa: the individual SP cell amplifications display the same overall transcriptional profile as the amplifications performed in a larger number of cells; the same applies to the SCE amplifications (for individual SCE profiles, see Figure S3). (B) The variance of single-cell amplifications (SC) is higher than SCE. The figure shows the standard deviation (SD) of the genes from SC and SCE experiments. The box plot shows the inter-quartile range and median of the SD values. For visual clarity, outlier values are not shown. The median standard deviation is higher in the single cell experiments, suggesting clear evidence for biological variation among the single cells. (C) Variance component analysis shows that a significant proportion of genes has a large component of biological variance. The graph represents the density of genes plotted against the ratio between the biological variance estimate and the total variance (the sum of the biological and technical variances). This ratio varies from 0 (all the variability in gene expression levels across single cells is attributable solely to technical variability) to 1 (all the variability is due entirely to biological variability). For the majority of genes, there is little or no biological variance. However, there is a sizeable share of genes with expression levels that exhibit extreme biological variance. The area under the curve indicates that the percentage of genes in which at least half of their variability is explained by biological variation is approximately 44%.

We next applied this amplification and analysis strategy to 12 individual SP cells directly sorted into wells of a 96-well plate. For these experiments, the SP cells from the lowest tip that were also Sca-1^+^ and Gr1^−^ were sorted, to ensure the greatest possible level of functional homogeneity. When we analyzed the expression levels of the 800 most discriminative genes between SP and CD8 (i.e., those with an associated *p* < 0.005), there was, in general, greater variability among single cells than among 10-cell pools. However, the amplified transcription profile maintained an accurate representation of the transcriptional profile differences between distinct populations. This can be appreciated in Figures 2A and S3, where we can see that genes that are expressed at higher levels in CD8 T cells (as determined by our prior analyses) tend to be present in lower levels in each of the single cells, and vice versa. Thus, the overall genetic expression data are not affected and transcriptional representation is maintained.

The variability observed among single SP cell transcription profiles could be due to biological differences that exist between individual stem cells, reflecting true gene expression differences between individual stem cells. Alternatively, technical differences in the cDNA preparation, PCR amplification, or chip hybridization of each cell could account for some or all of the variation. In order to determine exactly what proportion of variation was due to biological versus technical causes, we performed the same amplification and detection procedures in parallel on RNA from 5 single-cell-equivalents (SCE). These SCE were made by sorting 40 SP cells into 40 times the amount of lysis buffer and aliquoting the cell lysate so that each amplification and hybridization reaction was performed with a volume and quantity of RNA equivalent to that of a single cell. This effective pooling of the RNA should allow us to measure variation originating in the methodology. Single-cell amplifications contain both biological and technical variability, whereas SCE amplifications should only represent technical variation. Using the ratio of the SCE variances to the single cell observed variances we determined initially a conservative estimate of the minimal proportion of variation that can solely be attributed to biological differences for each gene. The overall normalized standard deviation of each gene for the SCE is indeed lower (average 0.106) than that of the individual stem cells (0.133), as it can be appreciated in Figure 2B. These results suggested that at least 20% of the variation in genetic expression levels must be exclusively due to biological fluctuation or differences in the transcripts of individual stem cells. Of note, when we compare the average expression of each gene between individual stem cells and SCE by applying the previously used statistical criterion (*p* < 0.05), only 18 genes are found to display a fold difference ≥ 1.6. Theoretically, since both samples represent the same population, there should be no differences in means between them. We believe that our findings are within the false discovery range expected by running so many simultaneous comparisons between a limited number of samples.

To further investigate our findings, we performed a variance component analysis on the expression of each gene represented in the microarrays (Figure 2C). Through this methodology, we were able to conclude that, although most variation in expression of the majority of the genes analyzed is attributable to technical factors, in a significant proportion of genes this is not the case. Instead, true biological variability accounts for the differences in expression levels observed among single cells. Specifically, the percentage of genes in which at least half of their variability is explained by biological variation is approximately 44%. Again, this establishes that a highly phenotypically homogeneous population of stem cells demonstrates real heterogeneity at the transcriptional level.

### Single-Cell Expression Analysis of Variability

In order to further clarify the finding of high variability in expression levels of single cells, we decided to take a closer look at the behavior of genes that have been previously associated with stem cell biology. The RMA software provided us with expression levels for every gene in each cell analyzed on the array (with a theoretical distribution from zero to plus infinity), but did not establish a threshold for actual “presence” or “absence” of a transcript in a particular sample. On the other hand, the Affymetrix microarray analysis algorithm (MAS5) classifies genes as “present” (P), “marginally present” (M), or “absent” (A) according to their expression levels in a single array based on the relative intensities of the perfect match and mismatch probes used to detect a particular transcript. We used these definitions of presence (P or M) and absence (A) to tentatively identify which genes were being transcribed above background levels at the time the GSC RT-PCR was performed.

**Figure 3:**
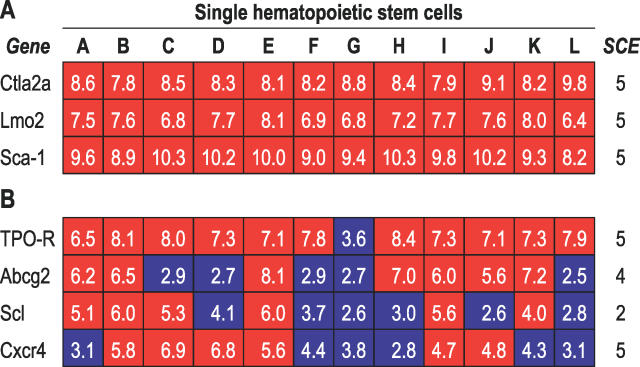
Expression Levels of Genes Previously Associated with HSC Genetic Profile Each single SP cell amplification experiment is represented by a different column (A–L). Genes given a P call by MAS5 are represented in red. Otherwise, they are shown in blue. The white numbers in the boxes represent the logged (base 2) gene expression levels calculated by RMA. The SCE column summarizes the number of P calls across five single-cell-equivalent (see text for explanation) amplification reactions. (A) Genes consistently present in all single cell amplifications performed using GSC RT-PCR followed by oligonucleotide array analysis. (B) Genes detected less frequently than expected. This can potentially be due to technical limitations or true biological variability among cells.

We found that several genes previously described in association with HSC populations, such as *Sca-1,* LIM domain only 2 *(Lmo2), Ctla-2a,* and thrombopoietin receptor *(TPO-R),* were indeed found to be present by our method in each of the single stem cells studied. However, to our surprise, some genes previously connected to HSC (such as *Flk2, Abcg2,* and *CXCR4*) appeared to be expressed above background in only a limited number of single cells. In general, the former genes tended to have low variance between cells, but the latter showed high variability (Figure 3A and 3B). Moreover, c-kit had consistently an “absent” call across almost all of the single-cell experiments.

To validate the differences observed between single cells, we assessed gene expression in single HSC using individual single-cell Q-PCR. Genes with high and low variance, as well as high and low expression were chosen. Gene expression patterns of genes considered to be “present” (i.e., genes with P call) or “marginally present” (i.e., genes with M call) by microarray analysis showed similar patterns of expression by Q-PCR (Table 3). For example, as predicted by our microarray experiments, *Ctla-2a* and *Lmo2* were both found to be expressed on all 12 single-cell microarrays and in all single-cell Q-PCR experiments. Moreover, *Lyl1* and *Scl* were found in approximately half of the single-cell Q-PCR amplifications, similar to the rate identified by our GSC RT-PCR combined with microarray strategy. In contrast, c-kit, which was considered to be “absent” on 11 single cell microarrays and “marginally present” on one of them, was shown to be expressed in 53% of HSC tested by real-time RT-PCR, indicating that lack of detection on a microarray with our method does not necessarily represent true absence of mRNA expression. As expected, Q-PCR is as, or more sensitive, than microarray detection. A few instances in which Q-PCR seems to be less sensitive, such as for *Bmp1* and *Csnk2a2,* could be related to non-specific cross-hybridization during microarray incubation.

**Table 3:**
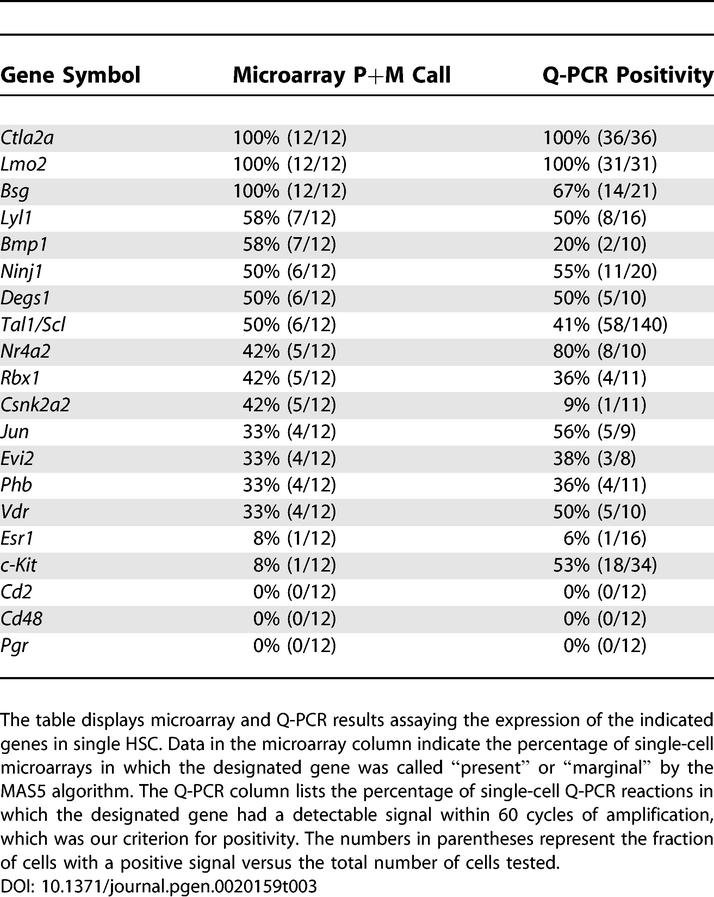
Q-PCR Validation of Single-Cell Microarray Data

**Figure 4:**
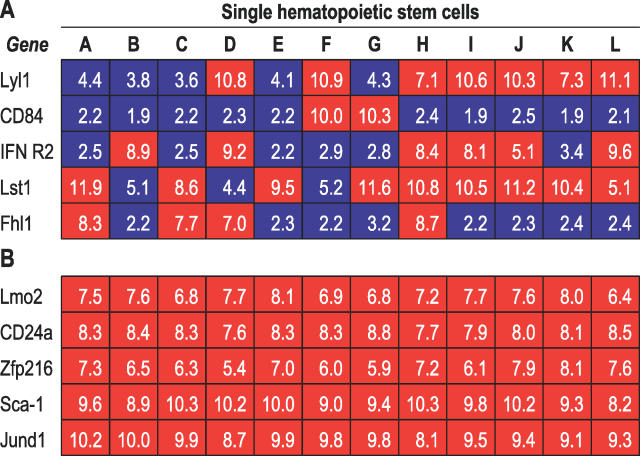
Selected Genes Consistently Expressed in SCE (Low Variance of Expression Levels), but Displaying High or Low Variability in Single SP Cells Genes with low variance in the SCE are likely to be detected accurately by our amplification procedure, since a fraction equivalent to one cell of the same pool of mRNA gives a consistent result across experiments: any variation detected in single cells should therefore reflect true differences in levels of gene expression. Some of these genes have low variation in single cells (B), while others (A) display different levels in distinct stem cells. The latter are likely to be responsible for heterogeneity in behavior of individual HSC (see Figure 3 for an explanation of the colors and numbers used).

As discussed above, variation in gene expression levels in our study can reflect either true biological variability, or technical variability. Our variance component analysis and our Q-PCR results suggest that a significant part of the variation in gene expression levels is real. In order to select which genes are reliably detected and biologically informative, several points need to be considered. Technical limitations can be due to poor amplification of some genes, or poor detection by non-optimal probes on the array; these genes will likely exhibit low expression levels with our technique even in the single-cell equivalents, and will have a small component of biological variability in the variance analysis. On the other hand, there are genes that display low variance across each of the single-cell-equivalent experiments, but still show high variability among single cells. In other words, these genes are detected at a similar level on identical mRNA samples (and consistently expressed on average in the stem cell population as a whole), but that they still show great variability between individual stem cells, which is detectable by our method and confirmed by Q-PCR. This second group of genes displays a large component of biological variance and is therefore the likely source of the biological variation observed in our data, making them potentially responsible for the functional variability of HSC (Figure 4 and Table S4).

To further characterize the biological differences between high and low biological variance genes, we performed a Gene Ontology (GO) analysis on the list of genes representing those two groups. For the purpose of the GO analysis, we defined high biological variance (HBV) genes as those having an estimated proportion of biological variance greater than 95% and with a P call in at least a third of the single cell arrays (467 probe sets); low biological variance (LBV) as those with less than 5% of biological variance and P calls in all single cell arrays (361 probe sets). We selected these cutoffs based on a density estimate of the proportion of biological variance across all genes as shown in Figure 2C. This density estimate shows clear bi-modality, and the 5% and 95% cutoffs are the approximate modes of the two peaks in the density estimate. Additionally, these two well-separated cutoffs seemed appropriate to determine two widely disparate variance based classes.

The GO categories significantly associated with each gene list defined GO signatures for HBV and LBV genes. Examples of genes and respective GO categories from each signature are represented in Table 4. The full GO analysis tables are available as Tables S7 and S8.

**Table 4:**
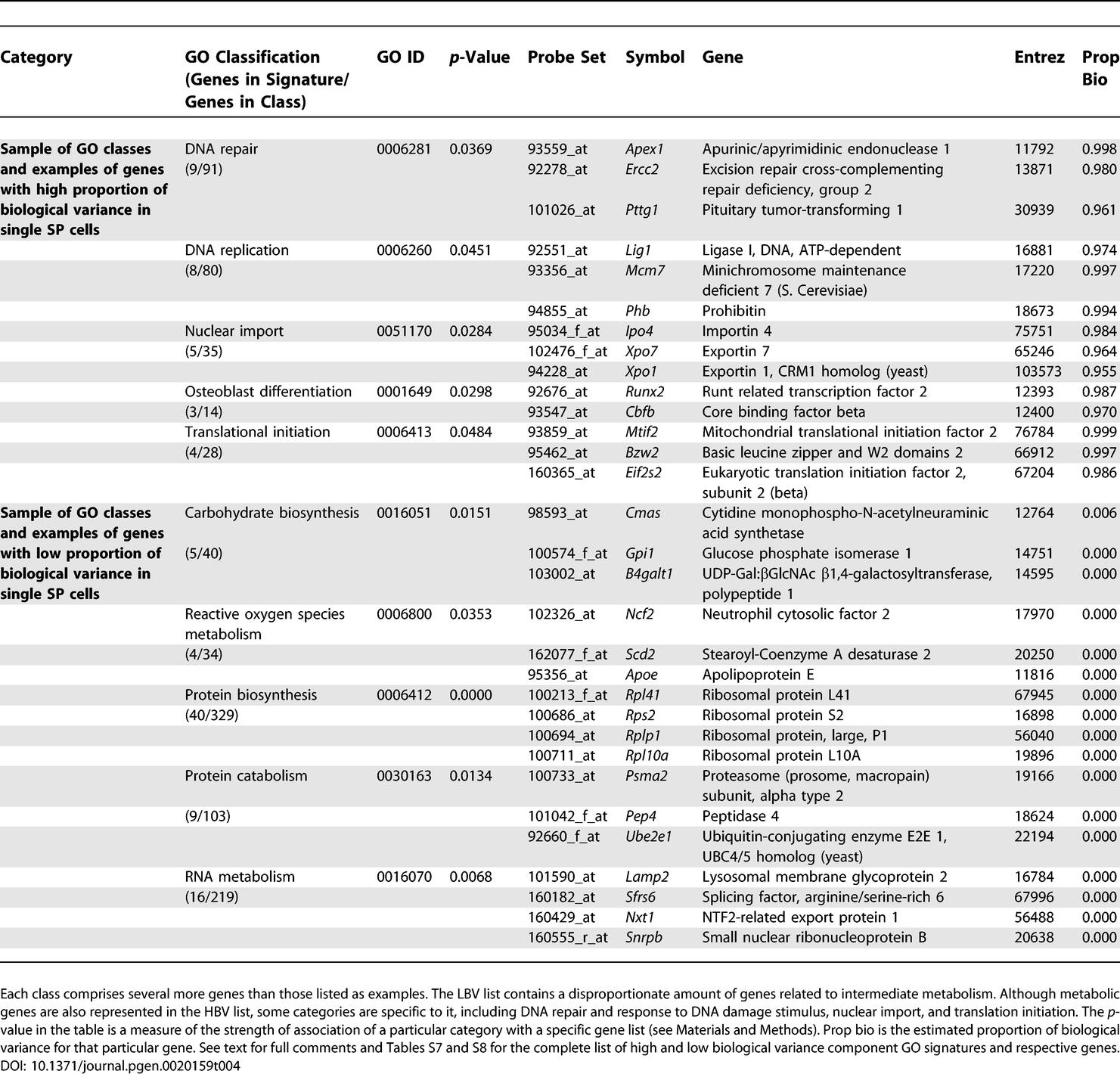
Sample of GO Classes with Examples of Corresponding Genes Found in GO Signature Tables for High and Low Biological Variance Component Genes

Salient features in the LBV group include an overrepresentation of genes involved in intermediate metabolism in the LBV signature. Furthermore, genes related to reactive oxygen species metabolism are also part of the LBV signature. This is of particular relevance since recent reports have highlighted the importance of these pathways in the self-renewal and control of life span of HSC [40,41]. Although the HBV signature also includes some metabolic genes, some categories appear to be specific to HBV, including genes related to DNA repair and replication, nuclear import, response to DNA damage stimulus and translational initiation (Table 4). Furthermore, a group of genes whose function has been traditionally attributed to osteoblast differentiation is significantly associated with HBV.

Examples of genes in the DNA repair and response to DNA damage categories include *Apex1* (apurinic/apyrimidinic endonuclease 1), *Ercc2* (excision repair cross-complementing repair deficiency, group 2), *Lig1* (ligase I, DNA, ATP-dependent), and *Pttg1* (pituitary tumor-transforming 1). Lack of *Apex1* has been involved in increased spontaneous mutagenesis in somatic and germ cells [42]. *Ercc2* has been previously described in the context of premature aging and decreased life span associated with increased sensitivity to oxidative damage [43]. Absence of *Pttg1* causes a variety of cell growth abnormalities [44]. *Runx2* (runt related transcription factor 2) and *Cbfb* (core binding factor beta) belong to the “osteoblast differentiation category”, but they may actually have a role outside this process. Both Cbfb and Runx family proteins have been shown to be required for normal maturation of hematopoietic cells as well as establishment of definitive hematopoiesis [45,46], and both have been associated with leukemogenesis [47]. Overall, it seems that whereas LBV is associated with a housekeeping function, HBV likely translates different states of readiness, switching mechanisms from a quiescent to a proliferative state or even control of early commitment steps.

In order to exclude any bias introduced in our results by the different localization of the probes along each gene, we also examined the extent to which probe location within transcripts was associated with the variance components. We found no difference in the median relative probe position between the groups of probe sets corresponding to HBV and LBV genes (Figure S4) demonstrating that HBV do not result from their probes being less 3′ biased than LBV. Finally, we took a closer look at the relationship between the intensity of the probes and the estimated proportion of biological variance. Because of their nature, HBV genes are likely to have on average lower expression levels: being variable means that sometimes there is no transcript present and this will bring the average expression levels lower. Therefore, better than comparing the distribution of average intensity of genes between HBV and LBV groups is to look at the distribution of the maximum intensity for each gene (since the theoretical maximal level of expression will never be lower than this value). When we do this, we can see that while it is true that HBV genes tend to have maximal expression levels slightly lower than those with LBV, both HBV and LBV gene lists have significant proportions of low expression level genes (Figure S5). This argues again that HBV does not result solely from differences in probe intensity (which would be disregarded by the SCE experiments). Instead, it suggests that HBV genes have more labile transcriptional levels, as opposed to highly expressed LBV genes.

## Discussion

Here, we have demonstrated that gene expression profiles of minimal numbers of cells, from a single cell to as few as ten cells, can be obtained using microarray technology. The method is both remarkably reproducible and accurate, generating profiles that can be used to cluster cell types with fidelity and to predict gene expression differences that can be readily validated using quantitative real-time PCR (Figure 1B). This methodology will be important for further investigating differences between these and other stem cell types, and understanding the role that micro-heterogeneity has in dictating biological function.

In contrast to previous efforts, we have studied the genetic expression of individual stem cells, instead of a population of stem cells [11–17]. A few authors have previously addressed the transcriptional profile of single cells of developing pancreas [24], metastatic foci [23], or adult hippocampus [25], but these studies have used platforms containing a limited number of genes. A previous publication has examined single olfactory neurons [26], which are very large cells containing substantial amounts of mRNA, but the expression levels of individual genes was not validated. We have demonstrated that the average relative expression levels determined by our method are accurate, examined the expression of tens of thousands of genes simultaneously and studied cells that have minimal cytoplasm and thus scant mRNA. Furthermore, by analyzing the transcriptional profile of stem cell equivalents, we were able to identify which genes are reliably measured using our method, which is unique to this study.

Similar to others, we have been unable to identify a group of genes that is specific for (i.e. only expressed in) HSCs. However, our work establishes that genetic expression differences within purified stem cell populations exist, which is critical when considering stem cell function. Our observations substantiate existing evidence suggesting variability in patterns of gene expression between individual cells in hematopoietic stem and progenitor cells and that have established the notion of multi-lineage gene priming [48–50]. The findings in these papers, which point to promiscuity in the expression of lineage-specific genes in early progenitors, support our results showing (possibly combined) early expression of myeloid and erythroid markers in the upper SP (which is lineage negative, by surface markers). However, it should be noted that the main focus of these above-referenced papers is that HSC or progenitors simultaneously express genes from different lineage-committed cells. The meaning of this finding and the potential reasons why this had not been seen previously by other investigators are discussed in the papers. Although some variability in expression of individual cells was noted, this was not the focus of the studies. Our work is also unique in that we have studied genetic expression differences at a much larger scale than single-cell RT-PCR for a limited number of genes.

When others have analyzed the genetic expression profiles of single cells from relatively homogeneous populations [24,51], while some of the transcripts studied were regularly detected in every cell, they also found consistent variation in expression levels of other genes. By studying the amplification of single cell equivalents, we have established that this cannot exclusively be ascribed to technical limitations. The use of SCE is superior to other possible strategies, such as studying a putative more homogenous population or establishing a threshold of detection for specific genes by spiking a known number of RNA molecules in our samples. On one hand, a common pool of RNA obtained by aliquoting a mixture of a few cells is more homogenous than even a cell line. On the other hand, a complex mixture of RNA will have amplification kinetics different from an isolated single molecule of RNA and any particular gene present in a complex mixture of RNA will have its own amplification kinetics. The comparison between single cells and SCE should tell us not only whether we are looking at real biological variation, but also what the inherent noise of the measurement is.

Our single-cell data corroborate previous observations that suggest that transcriptional activity is “quantal” and that, most likely, genes that are know to be active in a certain population may not necessarily be constantly transcribed at the same levels in every single cell [52–55]. Genetic expression fluctuation is indeed thought to occur in single cells from otherwise homogeneous populations and could constitute the basis for differences in stem cell fate and behavior. The differences that we observed between individual SP cells may be inherently stochastic or caused by micro-environmental differences in the stem cell niche, and may also dictate their differential response to activating stimuli. In turn, these may translate into diversity in the level of readiness of each stem cell or may predispose individual cells to differentiate toward distinct lineages.

In any case, one striking observation is that some genes exhibit very low variance between the single cells, while other genes exhibit high biological variance. The GO analysis indicates that the LBV genes primarily serve a house-keeping function, while the HBV group includes genes which seem to affect the state of readiness. This is likely related to the still poorly understood question of why only a few stem cells are activated at any given time. Some of the HBV genes identified here may dictate which cells are utilized first.

Although it is evident that we can detect biological differences, because of technical limitations, the expression of some genes will simply not be measurable using this technique. As previously observed in other studies of transcriptional profiling, some probe sets present on the Affymetrix microarrays tend not to be informative. These sets likely cross-react with repetitive sequences, recognize similar sequences in unrelated transcriptionally active genes or simply have poor hybridization kinetics. In addition, some genes are recognized on the array by multiple distinct probe sets, which in some cases give very different results. Because our method relies on the amplification of the 3′-most portions of transcripts, the minority of probe sets that are less 3′-biased (or well-distributed throughout the gene) may not detect the expression of some genes.

Expression of c-kit, negative on the array but variably positive in the Q-PCR, illustrates this point. However, c-kit also illustrates potential discrepancies between protein and mRNA expression, and especially between protein levels and transcript levels. We know from many other experiments that all SP cells purified as done here express abundant c-kit protein on their surface. Yet, that does not necessarily mean that this gene is being transcribed at high levels in every HSC. It is also possible that the low level of mRNA reflects a post-transcriptional regulation of c-kit, such as rapid degradation, that merits further study. One needs to keep in mind that gene expression levels calculated by microarray analysis are in fact measurements of levels of specific transcripts, which in turn depend both on gene expression activity and competing mRNA degradation.

In brief, some genes in the array will not be informative using our strategy. However, a considerable amount of them are reliably detected and we feel that, for the purposes of our experimental plan, the most informative candidates are those that have a consistent expression above background in the stem-cell equivalent experiments but variable detection between individual SP cells, even though this definition may overlook some important genes, such as *Bmi1,* which have moderate expression levels. In any case, based on our data, a significant proportion of genes on the array (almost half of those with a “present” call in at least a single cell) should be informative. Future studies of the role of genes differentially expressed among single stem cells will bring important insight regarding their physiology. Further refinements in microarray technology, PCR technique, amplification methods [56], and oligonucleotide chemistry will undoubtedly increase the detection accuracy by the GSC RT-PCR combined with oligonucleotide microarray strategy.

Our method will also be of use to derive gene expression profiles from limited number of cells that can be obtained using laser capture micro-dissection from tissue sections, enabling the definition of pre-disease gene profiles. Likewise, this methodology may be applicable to rare cell types that can only be identified currently by morphology and location, such as intestinal [57] stem cells and melanocytes [58]. Finally, single-cell expression profiling may be able to uncover genetic pathways in highly characterized biological systems such as Caenorhabditis elegans, where single genes are known to direct individual cell fate, but the down-stream consequences of master-gene expression are not fully understood.

## Materials and Methods

### Animals.

C57Bl/6 CD45.1 mice were used at 7–9 wk of age. Mice were bred and maintained on acidified water in the animal care facility at Baylor College of Medicine.

### Cell sorting.

For single or ten-cell experiments, whole bone marrow (WBM) was collected from the femora and tibiae of one mouse as previously described. For RNA isolation to use in Q-PCR, WBM was obtained from five mice and enriched for Sca-1 positive or CD3-positive cells using magnetic beads (autoMACS; Miltenyi Biotec, Sunnyvale, California, United States) conjugated with anti-biotin antibodies after incubation of the cells with biotinylated antibodies against the former molecules. The SP samples were initially incubated with Hoechst 33342 (Sigma, St. Louis, Missouri, United States) as previously described and stained with PE-conjugated anti-Sca-1 antibody (or PE-conjugated streptavidin) and FITC-conjugated anti-Gr-1 (Ly6G)/Ly6C antibody and sorted for cells positive for Sca-1 and negative for Gr-1/Ly6C in the SP region. The LSP was defined as the lower third of the SP region and the USP as the upper third of the SP region. The SP region was defined conservatively and did not include the upper shoulder of the tail. There was no overlap between USP and LSP. The CD8+ T cell samples were stained with FITC-conjugated anti-CD3 antibody (or FITC-conjugated streptavidin) and PE-conjugated anti-CD8 antibody and sorted for double positives (all antibodies from BD Pharmingen, San Diego, California, United States).

### Global single or minimal number cell RT-PCR.

We sorted single-cell or ten-cell samples using a MoFlo (Cytomation, Fort Collins, Colorado, United States) into individual wells of a 96-well PCR plate containing 4 μl of lysis buffer. For 100 μl of lysis buffer, we combined 76 μl of RNase free water, 20 μl of first strand buffer (20 mM Tris-HCl [pH 7.5], 100 mM NaCl, 0.1 mM EDTA, 1 mM DTT, 0.01% v/v NP-40, and 50% v/v glycerol), 1 μl of Prime RNase inhibitor (Brinkmann, Westbury, New York, United States), 1 μl of RNase Guard (Promega, Madison, Wisconsin, United States), 0.5 μl of NP-40, and 2 μl of a fresh 1/24 dilution of stock primer mix. The stock primer mix was prepared adding 1 μl of 100 mM dATP, 1 μl of 100 mM dCTP, 1 μl of 100 mM dGTP, 1 μl of 100 mM dTTP, and 2 μl of 500 μg/ml oligo-dT_12–18_ (Invitrogen, Carlsbad, California, United States) to 2 μl of RNase free water (Promega).

Following sorting, cells were incubated 1 min at 65 °C (to lyse the cytoplasmic membrane—the nuclear envelope remains intact—and denature the mRNA), 2 min at 25 °C (to allow the oligo-dT to anneal to the poly-A tails of the mRNA), and chilled on ice. Next, we added 0.5 μl of a 1:1 mix of M-MLV (200 U/μl) and AMV (2.5 U/μl) reverse transcriptases and we incubated the plate at 37 °C for 15 min and 65 °C for 10 min (to inactivate the enzymes).

After the RT, we added 4.5 μl of tailing buffer and 0.4 μl (25 U/μl) of terminal deoxynucleotidyl transferase (TdT) (Roche, Nutley, New Jersey, United States) and let this polyadenylation reaction run for 15 min at 37 °C, followed by an inactivation step of 10 min at 65 °C. The tailing buffer was made by mixing 400 μl of 5 × TdT buffer (500 mM potassium cacodylate [pH 7.2], 10 mM CoCl_2_, and 1 mM DTT) (Invitrogen) with 15 μl of 100 mM dATP and 585 μl of RNase-free water.

Next, we added 90 μl of PCR mix to the resulting products and performed the following PCR program: 25 cycles of 2-min denaturation at 94 °C, 2-min annealing at 42 °C, and 6-min (extending 10 s each cycle) extension at 72 °C. This was followed by, after adding extra 1 μl (5 U/μl) AmpliTaq (Roche), the same PCR program, without prolonging the extension time each cycle. For each PCR reaction, we mixed 53.5 μl of water, 10 μl of 10× PCR buffer II (100 mM Tris-HCl [pH 8.3] and 500 mM KCl), 10 μl of 25 mM MgCl_2_, 9 μl of 200 μM AL-1 primer, 4 μl of 100 mM dNTP, 1 μl of 5% v/v Triton X-100, 0.5 μl of 20 mg/ml bovine serum albumin, and 2 μl of AmpliTaq (5 U/μl). After the PCR, we ran 5 μl of each amplified product in a 1.5% agarose gel. If the amplification is successful, you should see a smear extending from around 300 bp to 1,200 bp (around 1 μg of DNA). However, frequently you can also see a smear in your negative (no cell) controls. This has been described previously [28] and is thought to result from bacterial contaminants present in the solutions. In order to identify successfully amplified cells, we prepared a Southern blot and probed it for a housekeeping gene *(GAPDH)*. See Protocol S1 for a comment on the GSC RT-PCR method.

### Target fragmentation.

The remaining poly-A cDNA was purified in a PCR cleanup column (Qiagen, Valencia, California, United States) and eluted in 50 μl of 10 mM Tris-HCl [pH 8.5]. We then used a Speed Vac machine (Jouan, Winchester, Virginia, United States) or Microcon-30 filter devices (Millipore, Billerica, Massachusetts, United States) to concentrate 25 μg of poly-A cDNA to 67.5 μl and added 14.2 μl of buffer mix. Buffer mix was prepared by combining 10.5 μl of One-Phor-All (Amersham, Piscataway, New Jersey, United States) buffer (100 mM Tris-acetate [pH 7.5], 100 mM magnesium acetate and 500 mM potassium acetate) to 6.6 μl of 25 mM CoCl_2_. We next added 0.5 U of DNase I (1 U/μl) (Invitrogen) and 9.5 μl of its respective 1× buffer (20 mM Tris-HCl [pH 8.4], 2 mM MgCl_2_, 50 mM KCl) and incubated the reaction for 3 min at 37 °C, followed immediately by placement in a boiling water bath for 15 min [33,34]. Adequate fragmentation of the cDNA should generate segments averaging 50 bp. The optimal concentration of DNase I and digestion time have been titrated for our reaction, by running a digested sample aliquot on a denaturing 10% PAGE together with an appropriate molecular weight marker.

### Target labeling.

In order to end-label the cDNA fragments with biotin, we next added 3.12 μl of 1 mM N^6^-biotinylated ddATP (PerkinElmer, Boston, Massachusetts, United States) and 4.25 μl of TdT (15 U/μl) (Invitrogen) and incubated the reaction at 37 °C for 2 h [33,34].

### Microarray incubation.

The biotin-labeled fragments were used directly as targets for GeneChip Murine Genome U74A version 2 microarrays (Affymetrix, Santa Clara, California, United States) according to the Affymetrix standard protocol, but scaling down reactions to a final hybridization mixture volume of 250 μl. After hybridization with 200 μl of this solution (equivalent to 20 μg of cDNA), the arrays were incubated according to the Affymetrix protocol (antibody amplified) with phycoerythrin-conjugated streptavidin.

### Microarray analysis.

The raw intensity data for each probe were collected with Microarray Suite version 5.0 software, MAS5, from Affymetrix (http://www.affymetrix.com). Expression levels for each of the genes represented in the array was computed in the R statistical programming environment using the Bioconductor implementation of the RMA method (http://www.bioconductor.org). The primary data used in our work are compiled in Table S5. Analysis to compute linear model and ANOVA results was also performed in R using the limma package and using the mixed model methods in the limma package to account for technical replication. As well, the limma based empirical Bayes methods were used to enhance the single gene T-statistics. A gene was considered to be differentially expressed between groups if a Student's t-test statistic had an associated *p* < 0.05. In generating tables of differentially expressed genes, we used unadjusted *p*-values, but we provide both the unadjusted and the adjusted *p*-values (calculated with the Benjamini-Hochberg linear step up procedure) in Tables S1 and S3. The use of the t statistic is more adequate for our type of experiments than the application of an absolute threshold for fold change. On the one hand, genes that have small fold changes but consistent levels (i.e., low variance) within different groups of samples, will be tagged as differentially expressed. On the other hand, genes that display high variability, either reflecting true biological fluctuations or limitations of the amplification method, will not be considered to be different between groups because their variances will be too high. Consequently, several false discoveries of genetic expression differences between groups will be avoided.

### Q-PCR validation for population expression levels.

We designed primers for a total of 22 randomly selected genes: seven whose expression levels were significantly higher in T cells, eight in SP cells, and seven not statistically different (Table S2). We isolated total RNA from a minimum of 200,000 SP or CD8 cells, pooled from different sorting experiments, using the RNeasy kit (Ambion, Austin, Texas, United States). The RNA was digested with DNAse I (Invitrogen) and resuspended in RNase free water (Promega) at a final concentration corresponding to 20,000 cells per μl. Each 10 μl of RNA solution was incubated with 1 μl of 500 μg/ml oligo-dT_12–18_ (Invitrogen) and 1 μl of 10 mM dNTP (Invitrogen) at 65 °C for 5 min. We then added 4 μl of first strand buffer (Invitrogen), 2 μl of 1 M DTT (Invitrogen) and 1 μl of RNAse out (Invitrogen) and kept the mixture at 42 °C for 2 min. Finally, we performed a reverse transcription reaction by adding 1 μl of SuperScript (Invitrogen). No-RT controls were done in parallel for each initial RNA sample and used to assess contamination with undigested DNA. The cDNA obtained had a final concentration corresponding to 10,000 cells per μl.

Each Q-PCR reaction was performed by mixing 5 μl of 10× PCR buffer with SYBR Green (PerkinElmer), 6 μl of 25 mM MgCl_2_, 4 μl of 12.5 mM dNTP, 0.25 μl of AmpliTaq (5 U/μl) (PerkinElmer), 3 μl of 5 mM forward primer, 3 μl of 5 mM reverse primer, 0.25 μl of cDNA template (equivalent to approximately 2,500 cells), and 28.5 μl of RNAse free water (final reaction volume 50 μl).

The PCR program used was the following: 2-min warm-up at 50 °C; 10-min denaturation at 95 °C; 45 cycles of 1-min annealing and extension at 60 °C and 15-s denaturation at 95 °C; 20-sec annealing at 60 °C, and 20-min ramp up to 95 °C (for melting curve acquisition). The reactions were performed in an ABI 7900HT (Applied Biosystems, Foster City, California, United States).

No-template controls were always performed for each primer pair and these PCR reactions were consistently negative for the presence of amplicons. All reactions were run in duplicate and the mean threshold cycle (C_T_) was calculated for each pair of reactions performed for each of the two populations (C_T_^SP^ and C_T_^CD8^). These were normalized for beta-actin levels in each RNA sample (C'_T_^SP^ = C_T_^SP^ – A_T_^SP^ and C'_T_^CD8^ = C_T_^CD8^ – A_T_^CD8^, where A_T_ is threshold cycle for beta-actin in a particular sample). The log fold change for each gene is given by LFC_QPCR_ = C'_T_^SP^ – C'_T_^CD8^. Every selected gene was tested in two independent RNA samples obtained from each population. An average log fold change (Log QPCR) between the two populations was obtained for each of the 22 genes studied.

A parallel value was calculated from the RMA data obtained from ten-cell microarray experiments. The average expression levels for six SP samples (performed in duplicate) and three CD8 samples (idem) were calculated (E'^SP^ and E'^CD8^). Since RMA expression levels are in a logarithmic scale, the log fold change is given by LFC_RMA_ = E'^SP^ – E'^CD8^ (Log RMA). Corresponding log fold changes (Log QPCR and Log RMA) for each gene were plotted in the same graph. A linear regression model was fitted to the values obtained.

### Q-PCR validation for single-cell experiments.

We sorted single HSC into single wells of a 96-well plate containing 4 μl of the lysis buffer used in our GSC RT-PCR reactions. We included both negative and positive controls on each plate: wells containing only lysis buffer (no cell) were used as negative controls, and wells containing lysis buffer plus 25 stem cells were used as positive controls. A single gene was analyzed per 96-well plate to minimize cross-contamination. Each 96-well plate was heated at 65 °C for 1 min and 25 °C for 2 min. We performed reverse transcription reactions by adding 0.5 μl of Superscript II reverse transcriptase (Invitrogen) followed by incubation of the plate at 42 °C for 60 min.

Following reverse transcription, cells were assayed for gene expression using multiplexed Q-PCR, which simultaneously detected 18S rRNA and our gene-of-interest. Each reaction was performed by mixing 25 μl TaqMan universal PCR master mix (Applied Biosystems), 2.5 μl of 18S rRNA endogenous control (VIC/MGB), 2.5 μl of gene-of-interest TaqMan gene expression assay (FAM/MGB), and 16 μl of nuclease-free water. The TaqMan gene expression assays (Applied Biosystems) used included Mm00484032_g1 *(Ctla2a),* Mm00493153_m1 *(Lmo2),* Mm00493153_m1 *(Lyl1),* Mm00441665_m1 *(Tal1),* and Mm00445212_m1 *(c-Kit).* We utilized the following cycling parameters: stage 1, 50 °C for 2 min; stage 2, 95 °C for 10 min; and stage 3, 94 °C for 15 s and 60 °C for 1 min. Stage 3 was repeated for a total of 60 cycles. A single cell Q-PCR was considered successful when the C_T_ for 18S was within the range of 26–30; the average 18S signal for all tested single cells was 28.5, while the average 18S C_T_ for negative control wells was 34. A gene was considered to be expressed when it had a detectable signal within 60 cycles of amplification. None of the negative control wells gave rise to a positive signal for the genes studied.

### Variance component analysis.

We used maximum likelihood to estimate the technical and biological contribution to the variance of each gene with at least one P call in a single cell (6,675 genes) using all 17 data points from the single-cell and single-cell-equivalent data. We assumed a Gaussian model for the expression measures and considered the total variance for single cell experiments to be the sum of independent technical and biological components, while for the single cell equivalent data we assumed only a technical component. We further supposed that the magnitude of the technical variance was the same between the single cell and single cell equivalent experiments. We used numerical methods to maximize the log-likelihood function for each gene using the *nlm* procedure in R (http://www.r-project.org) to directly maximize the function.

### GO analysis.

The GO analysis strategy used has been previously described [16]. Briefly, to assess the significance of gene counts at each GO category for a particular gene list, these were compared to corresponding counts for the whole array. The probability of a count of k genes in a GO node at some node of the G hierarchy was modeled according to the hypergeometric probability law P(X=k) = B(C,k)·B(L-C,n-k)/B(L,n), where B(x,y) stands for the binomial coefficient for x choose y. C is the total number of genes in the array annotated to the GO node being considered, L is the number of genes in the array annotated to all nodes, and n is the number of genes in a gene list annotated to a GO term. The one-sided *p*-value for the node under consideration is obtained by summing the probabilities obtained from the formula for all X values from k to n. The GO signature for a particular gene list is the subset of all GO categories represented in that particular list which have an associated unadjusted *p*-value less than 0.05.

## Supporting Information

Figure S1 GSC RT-PCR Combined with Oligonucleotide Microarray Analysis In our experiments, one or a few cells were lysed in a first strand buffer and a short reverse transcription was performed (A), generating limited-size cDNAs that represent the 3′-most few-hundred nucleotides of every mRNA molecule in solution. These products were then polyadenylated (B) and amplified in a PCR reaction primed by an oligonucleotide containing a poly-T tract (C–F). After random digestion with DNase I into fragments averaging 50 bp, these targets were end-labeled with biotinylated ddATP (G) and incubated with Murine Genome U74A version 2 microarrays according to the standard protocol (Affymetrix). For details, see Protocol S1.
(93 KB PDF)

Figure S2 The Transcriptional Representation Obtained by GSC RT-PCR and DNase I Fragmentation of cDNA is Comparable between Replicates Three representative samples obtained from LSP (LA), USP (UN), and CD8 cells (XB) are depicted. For each replicate pair, the expression levels for each gene are plotted against each other (upper row). Corresponding M-A plots are also shown (bottom row). The distribution around a line with slope equal to one demonstrates that the expression levels obtained are approximately the same. Each of the replicate pairs used in our experiments has a correlation value between 0.97 and 0.99.
(2.6 MB PDF)

Figure S3 Genetic Representation of Single-Cell Amplifications The same list of genes used in Figure 4 was applied to generate the heat map. Expression levels are normalized to 1 (white). Red genes are over-expressed and blue genes under-expressed in that sample. Each individual SCE sample is represented instead of the average of their expression levels.
(97 KB PDF)

Figure S4 Position Analysis of High and Low Biological Variability Genes in Single SP Cells We performed a position analysis to compare high biological variance and low biological variance results and identified 467 high variance probe sets with present calls in at least four single cells and more than 95% of total variance estimated to be biological and 361 probe sets with present calls in at least 12 samples and having less than 5% of total variance estimated to be biological. The median probe position for each probe set was normalized to the size of the gene, with 1 representing the 3′ end, and 0 the 5′ end (horizontal axis). Density is a measurement of the relative amount of probe sets for each median position (vertical axis). A Wilcoxon signed rank test shows no significant difference in the median relative probe position between the groups of probes sets.
(16 KB PDF)

Figure S5 Intensity Analysis of High and Low Biological Variability Genes in Single SP Cells The same gene sets used in Figure S4 were used for the analysis. The relative amount of probe sets (density) is plotted against the maximum expression level for a particular gene in the set of single cells studied (as determined by the RMA algorithm). While genes with high biological variance tend to have slightly lower maxima, the distribution is similar between genes with high biological variance and with low biological variance, with both groups having a similar low intensity tail.
(15 KB PDF)

Protocol S1 Detailed Biological Methods (64 KB DOC)

Table S1 Differentially Expressed Genes between SP Cells and CD8 T Lymphocytes (608 KB XLS)

Table S2 List of Random Genes Selected for Q-PCR Validation (20 KB XLS)

Table S3 Differentially Expressed Genes between LSP Cells and USP Cells (113 KB XLS)

Table S4 Variance Component Analysis for Genes with Four or More “Present” Calls in Single Cells (3.0 MB XLS)

Table S5 RMA Expression Levels in Each of the Samples Amplified with GSC RT-PCR Followed by Oligonucleotide Microarray Analysis (9.2 MB XLS)

Table S6 MAS Calls in Each of the Samples Amplified with GSC RT-PCR Followed by Oligonucleotide Microarray Analysis (7.9 MB XLS)

Table S7 GO Classification Tables for High and Low Biological Variance Genes (2.7 MB XLS)

Table S8 GO Signature Tables for High and Low Biological Variance Genes (197 KB XLS)

### Accession Numbers

The Entrez Gene (http://www.ncbi.nlm.nih.gov/entrez/query.fcgi?db=gene) accession numbers for the genes and gene products discussed in this paper are *Cd3g* (12502), *Cd3d* (12500), *Cd3e* (12501), *Cd3z* (12503), *Zap70* (22637), *Cd8a* (12525), *Cd8b1* (12526), *Tal1/Scl-1* (21349), *Ctla2b* (13025), *Abcb1b/Mdr1* (18669), *Abcg2* (26357), *Twsg1* (65960), *Zfx* (22764), *E2f1* (13555), *Ccna2* (12428), *Hba-a1* (15122), *Hbb* (15127), *Hbb-b2* (15130), *Cd14* (12475), *Ly6c* (17067), *Ly6a/Sca-1* (110454), *Lmo2* (16909), *Ctla2a* (116913), *Mpl/TPO-R* (17480), *Flt3/Flk-2* (14255), *Cxcr4* (12767), *Lyl1* (17095), *Apex1* (11792), *Ercc2* (13871), *Lig1* (16881), *Pttg1* (30939), *Runx2* (12393), and *Cbfb* (12400).

## Abbreviations

GO: gene ontology
GSC RT-PCR: global single-cell RT-PCR
HBV: high biological variance
HSC: hematopoietic stem cell
LBV: low biological variance
LSP: lower SP
Q-PCR: quantitative PCR
RMA: robust microchip analysis
SCE: single-cell-equivalents
SP: side population
USP: upper SP
