# Correction: Evidence for Diversity in Transcriptional Profiles of Single Hematopoietic Stem Cells

**DOI:** 10.1371/journal.pgen.0020201

**Published:** 2006-11-24

**Authors:** Carlos A Ramos, Teresa A Bowman, Nathan C Boles, Akil A Merchant, Yayun Zheng, Irma Parra, Suzanne A. W Fuqua, Chad A Shaw, Margaret A Goodell

In *PLoS Genetics,* vol 2, issue 9: DOI: 10.1371/journal.pgen.0020159

In the Materials and Methods section, under “Global single or minimal number cell PR-PCR”, the first-strand buffer was reported incorrectly as “20 mM Tris-HCl [pH 7.5], 100 mM NaCl, 0.1 mM EDTA, 1 mM DTT, 0.01% v/v NP-40, and 50% v/v glycerol.” The correct (5x) buffer should be: 250 mM Tris-HCl [pH 8.3]; 375 mM KCl; 15 mM MgCl_2_.

This buffer was also reported incorrectly in the Protocol S1. The second sentence under the heading “Global Single or minimal number Cell RT-PCR (GSC RT-PCR)” on the second page should be: “For 100 μL of lysis buffer, we combined 76 μL of RNase free water, 20 μL of first strand buffer 5x (250 mM Tris-HCl [pH 8.3], 375 mM KCl, 15 mM MgCl_2_), 1 μL of Prime RNase inhibitor (Brinkmann, Westbury, NY, USA), 1 μL RNase Guard (Promega, Madison, WI, USA), 0.5 μL of NP-40 and 2 μL of a fresh 1/24 dilution of stock primer mix.”

