# Supplementary figures and images for: Evidence for Diversity in Transcriptional Profiles of Single Hematopoietic Stem Cells
Source: PLoS Genet. 2006 Sep 29;2(9):e159. doi: 10.1371/journal.pgen.0020159 (PMC1584276; doi:10.1371/journal.pgen.0020159)

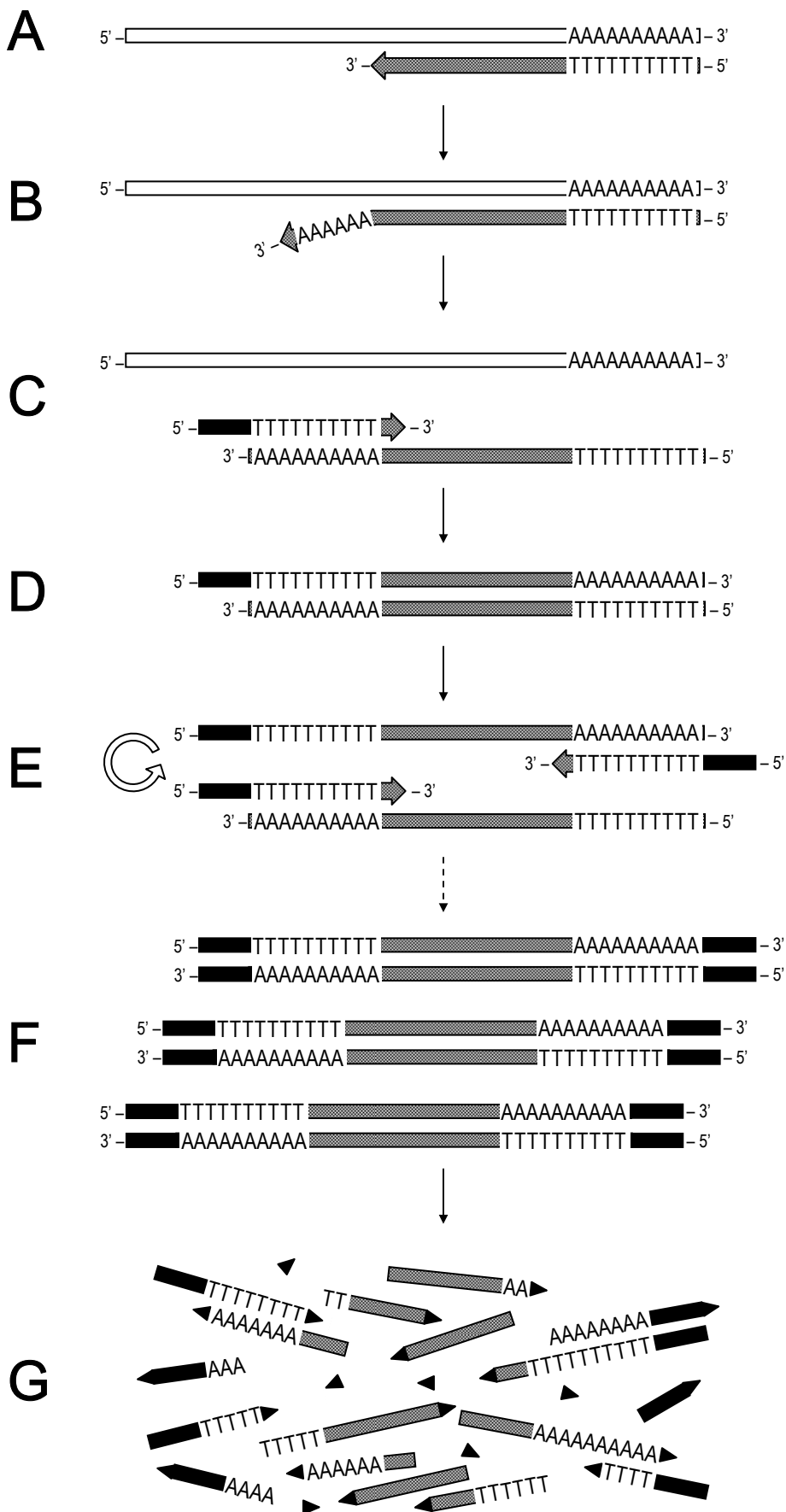

Supplement: Figure S1 — In our experiments, one or a few cells were lysed in a first strand buffer and a short reverse transcription was performed (A), generating limited-size cDNAs that represent the 3′-most few-hundred nucleotides of every mRNA molecule in solution. These products were then polyadenylated (B) and amplified in a PCR reaction primed by an oligonucleotide containing a poly-T tract (C–F). After random digestion with DNase I into fragments averaging 50 bp, these targets were end-labeled with biotinylated ddATP (G) and incubated with Murine Genome U74A version 2 microarrays according to the standard protocol (Affymetrix). For details, see Protocol S1. (93 KB PDF) [file pgen.0020159.sg001.pdf]

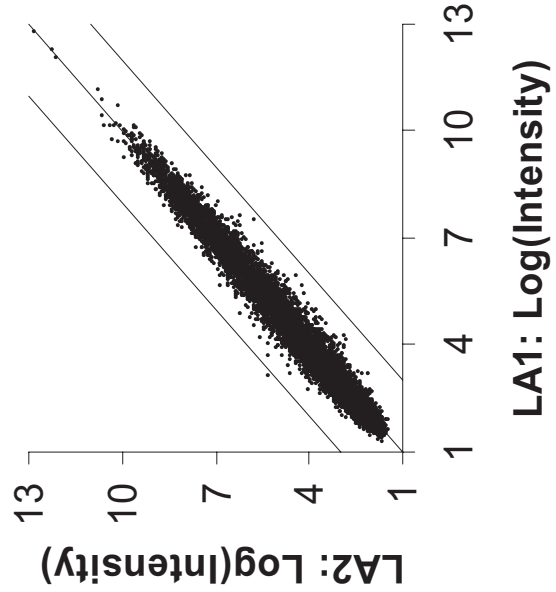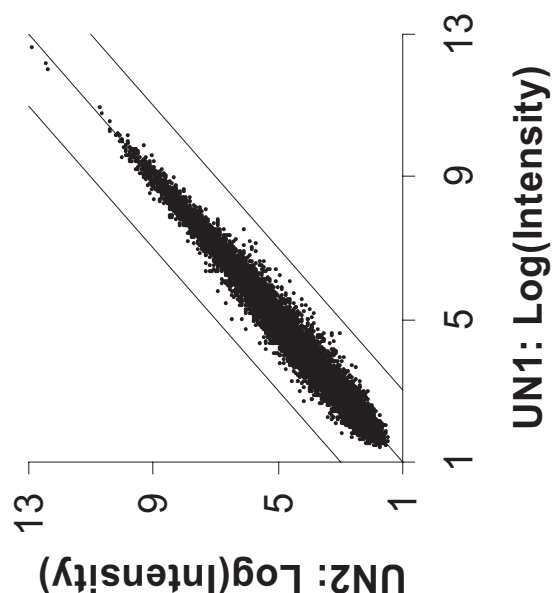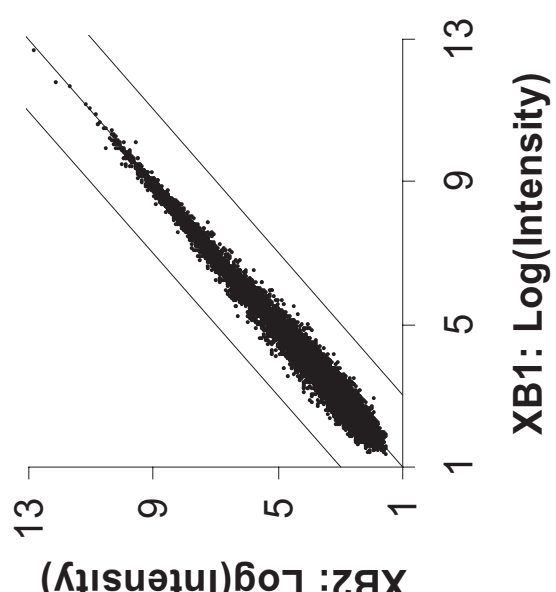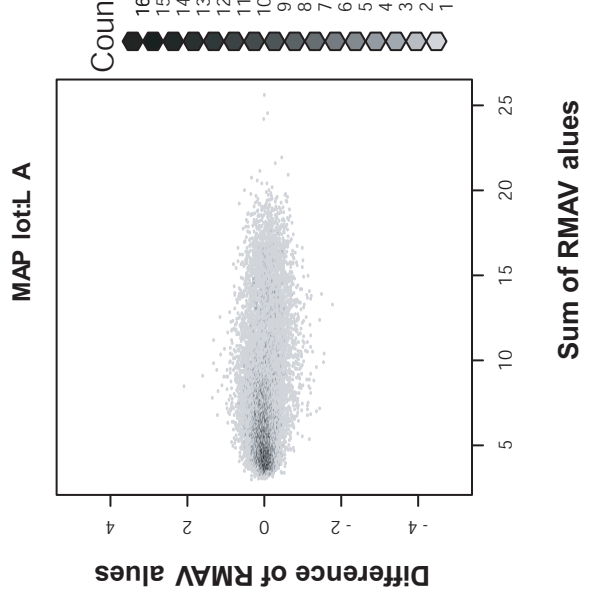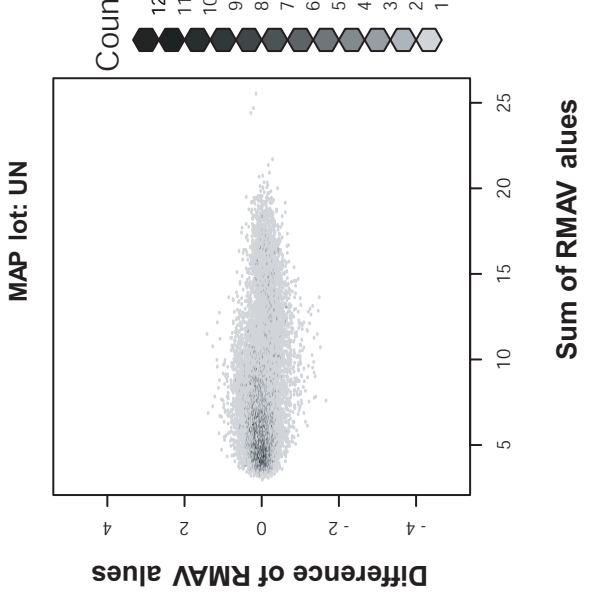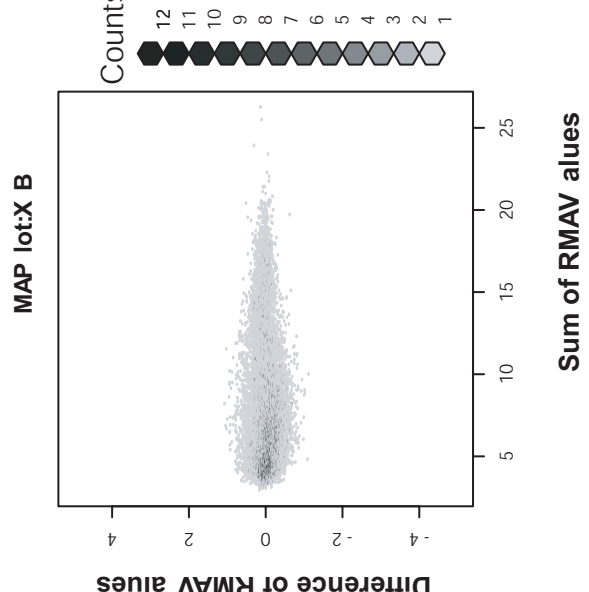

Supplement: Figure S2 — Three representative samples obtained from LSP (LA), USP (UN), and CD8 cells (XB) are depicted. For each replicate pair, the expression levels for each gene are plotted against each other (upper row). Corresponding M-A plots are also shown (bottom row). The distribution around a line with slope equal to one demonstrates that the expression levels obtained are approximately the same. Each of the replicate pairs used in our experiments has a correlation value between 0.97 and 0.99. (2.6 MB PDF) [file pgen.0020159.sg002.pdf]

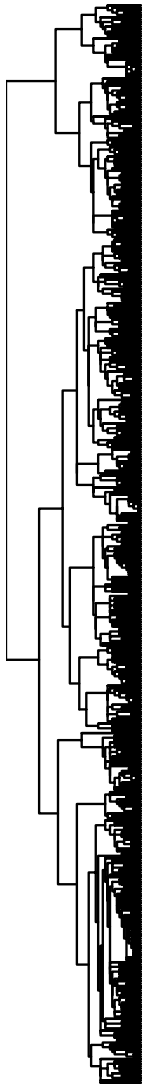

Single SP cells

SCE

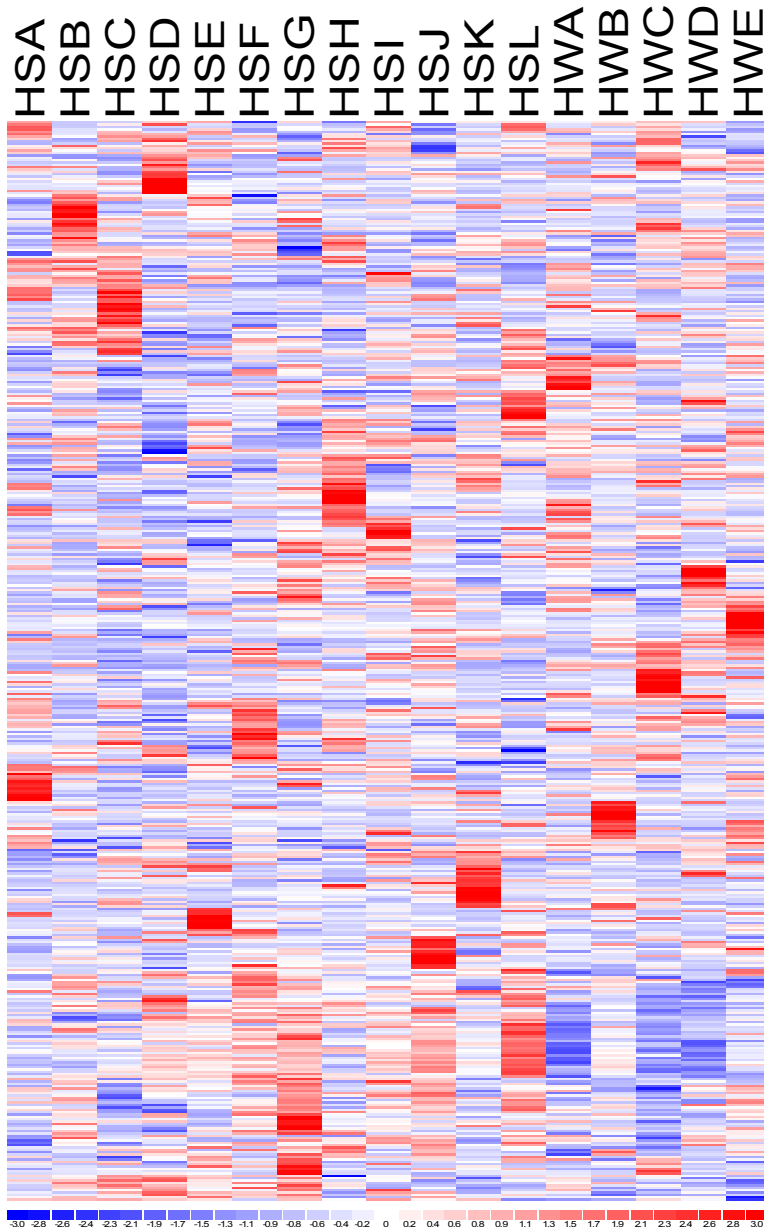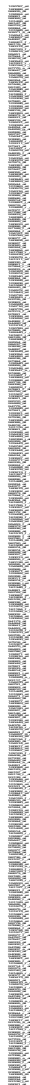

Supplement: Figure S3 — The same list of genes used in Figure 4 was applied to generate the heat map. Expression levels are normalized to 1 (white). Red genes are over-expressed and blue genes under-expressed in that sample. Each individual SCE sample is represented instead of the average of their expression levels. (97 KB PDF) [file pgen.0020159.sg003.pdf]

# Position Analysis

## high variance vs low variance probes

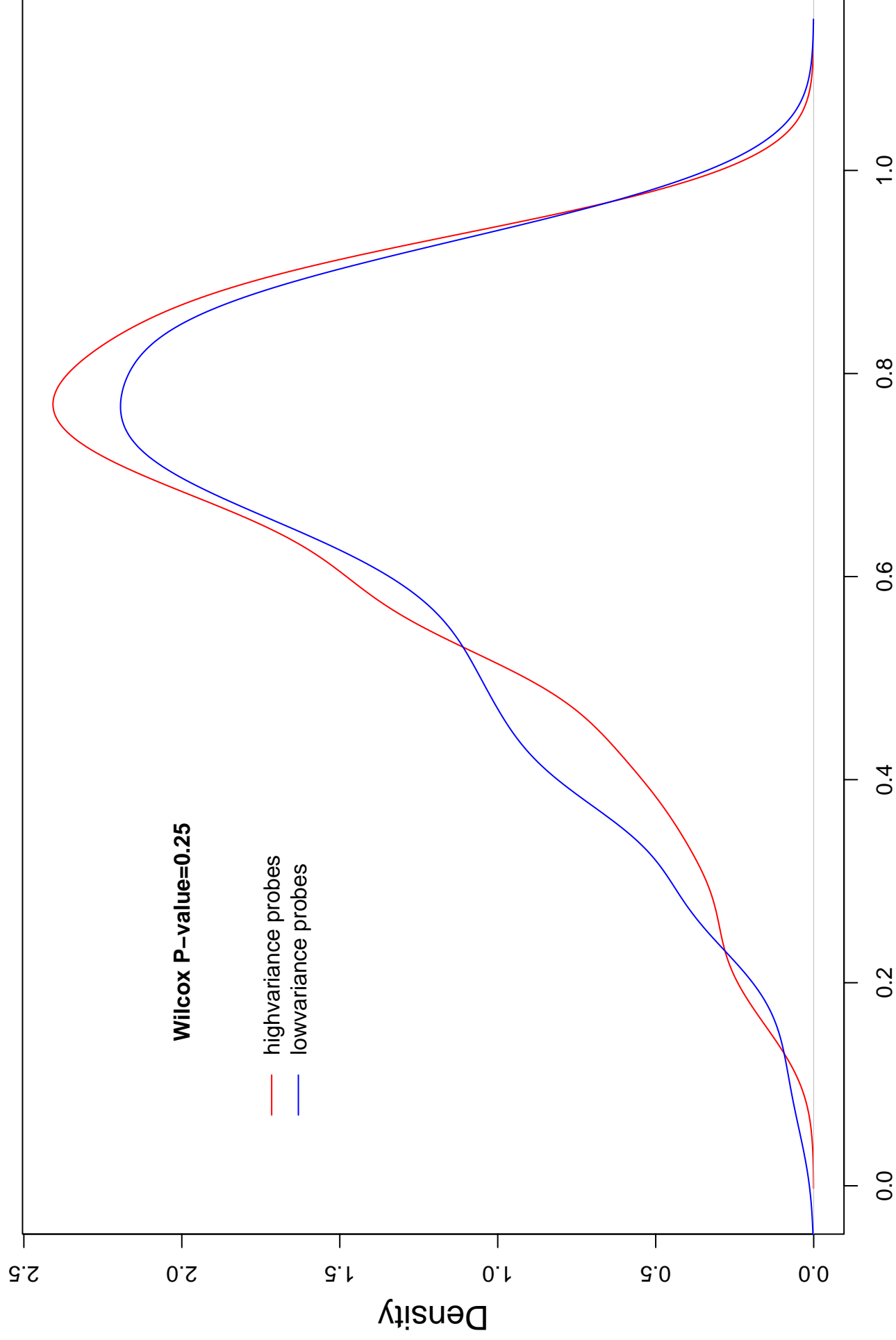

Supplement: Figure S4 — We performed a position analysis to compare high biological variance and low biological variance results and identified 467 high variance probe sets with present calls in at least four single cells and more than 95% of total variance estimated to be biological and 361 probe sets with present calls in at least 12 samples and having less than 5% of total variance estimated to be biological. The median probe position for each probe set was normalized to the size of the gene, with 1 representing the 3′ end, and 0 the 5′ end (horizontal axis). Density is a measurement of the relative amount of probe sets for each median position (vertical axis). A Wilcoxon signed rank test shows no significant difference in the median relative probe position between the groups of probes sets. (16 KB PDF) [file pgen.0020159.sg004.pdf]

# High Bio-variance vs Low Bio-Variance

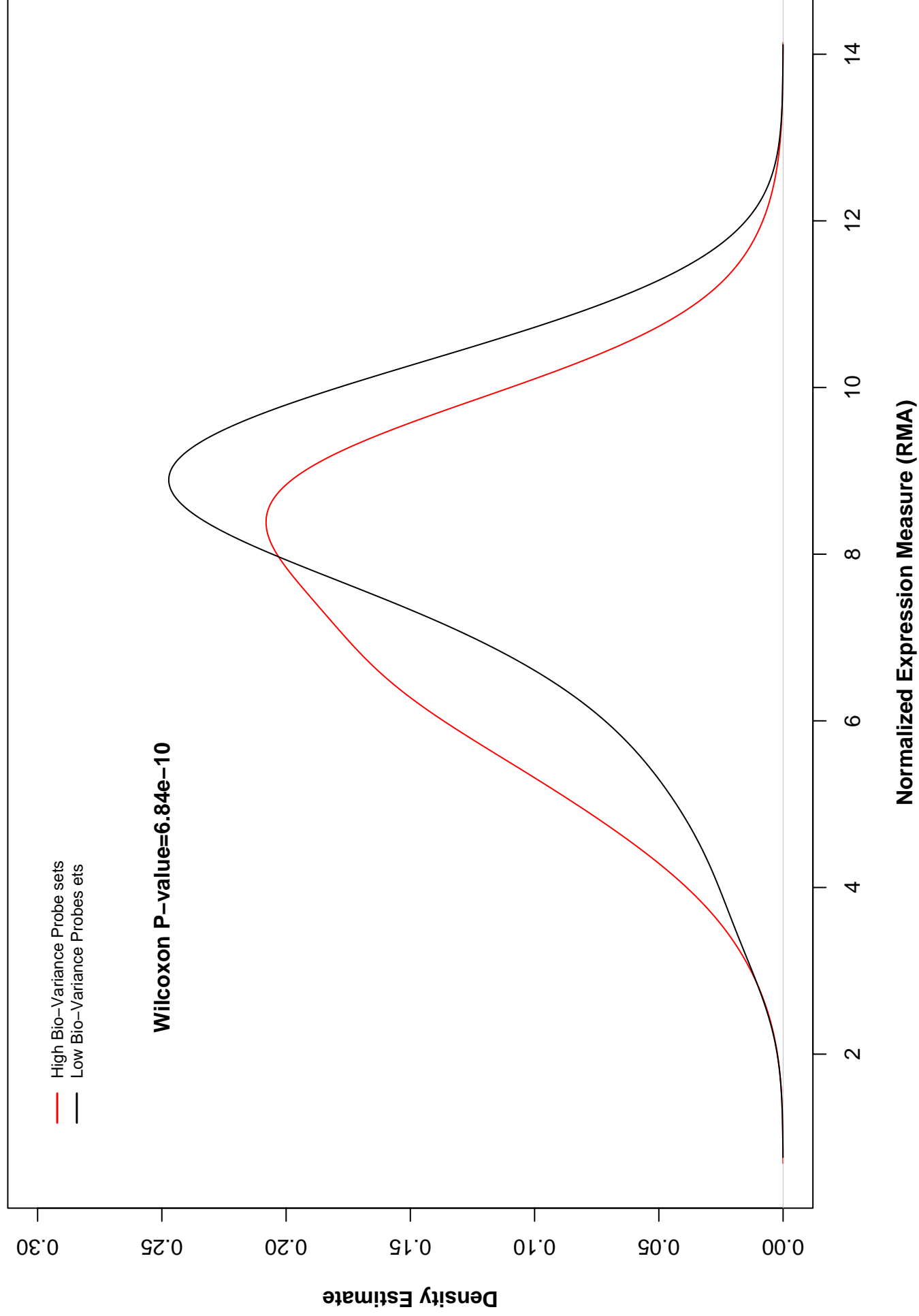

Supplement: Figure S5 — The same gene sets used in Figure S4 were used for the analysis. The relative amount of probe sets (density) is plotted against the maximum expression level for a particular gene in the set of single cells studied (as determined by the RMA algorithm). While genes with high biological variance tend to have slightly lower maxima, the distribution is similar between genes with high biological variance and with low biological variance, with both groups having a similar low intensity tail. (15 KB PDF) [file pgen.0020159.sg005.pdf]
