# Supplementary material for: Evidence for Diversity in Transcriptional Profiles of Single Hematopoietic Stem Cells
Source: PLoS Genet. 2006 Sep 29;2(9):e159. doi: 10.1371/journal.pgen.0020159 (PMC1584276; doi:10.1371/journal.pgen.0020159)
Supplement: Protocol S1 — (64 KB DOC) [file pgen.0020159.sd001.doc]

## Detailed Biological Methods

### Animals

C57Bl/6 CD45.1 mice were used at 7-9 weeks of age. Mice were bred and maintained on acidified water in the animal care facility at Baylor College of Medicine.

### Cell sorting

For single or 10-cell experiments, whole bone marrow (WBM) was collected from the femora and tibiae of one mouse as previously described. For RNA isolation to use in quantitative real-time PCR (Q-PCR), WBM was obtained from 5 mice and enriched for Sca-1 positive or CD3-positive cells using magnetic beads (autoMACS; Miltenyi Biotec, Sunnyvale, CA, USA) conjugated with anti-biotin antibodies after incubation of the cells with biotinylated antibodies against the former molecules. The SP samples were initially incubated with Hoechst 33342 (Sigma, St. Louis, MO, USA) as previously described and stained with PE-conjugated anti-Sca-1 antibody (or PE-conjugated streptavidin) and FITC-conjugated anti-Gr-1 (Ly6G)/Ly6C antibody and sorted for cells positive for Sca-1 and negative for Gr-1/Ly6C in the SP region. The lower SP (LSP) was defined as the lower third of the SP region and the upper SP (USP) as the upper third of the SP region. The SP region was defined conservatively and did not include the upper shoulder of the tail. There was no overlap between USP and LSP. The CD8+ T cell samples were stained with FITC-conjugated anti-CD3 antibody (or FITC-conjugated streptavidin) and PE-conjugated anti-CD8 antibody and sorted for double positives (all antibodies from BD Pharmingen, San Diego, CA, USA).

### Global Single or minimal number Cell RT-PCR (GSC RT-PCR)

We sorted single or 10 cells using a MoFlo (Cytomation, Fort Collins, CO, USA) into individual wells of a 96-well PCR plate containing 4 µL of lysis buffer. For 100 µL of lysis buffer, we combined 76 µL of RNase free water, 20 µL of first strand buffer (20 mM Tris-HCl pH 7.5, 100 mM NaCl, 0.1 mM EDTA, 1 mM DTT, 0.01% v/v NP-40 and 50% v/v glycerol), 1 µL of Prime RNase inhibitor™ (Brinkmann, Westbury, NY, USA), 1 µL RNase Guard™ (Promega, Madison, WI, USA), 0.5 µL of NP-40 and 2 µL of a fresh 1/24 dilution of stock primer mix. The stock primer mix was prepared adding 1 µL of 100 mM dATP, 1 µL of 100 mM dCTP, 1 µL of 100 mM dGTP, 1 µL of 100 mM dTTP and 2 µL of 500 µg/mL oligo-dT_12-18_ (Invitrogen, Carlsbad, CA, USA) to 2 µL of RNase free water (Promega).

Following sorting, cells were incubated one minute at 65°C (to lyse the cytoplasmatic membrane – the nuclear envelope remains intact – and denature the mRNA), 2 minutes at 25°C (to allow the oligo-dT to anneal to the poly-A tails of the mRNA) and chilled on ice. Next, we added 0.5 µL of a 1:1 mix of M-MLV (200 U/µL) and AMV (2.5 U/µL) reverse transcriptases and we incubated the plate at 37°C for 15 minutes and 65°C for 10 minutes (to inactivate the enzymes).

After the RT, we added 4.5 µL of tailing buffer and 0.4 µL (25 U/µL) of terminal deoxynucleotidyl transferase (TdT) (Roche, Nutley, NJ) and let this polyadenylation reaction run for 15 min at 37°C, followed by an inactivation step of 10 min at 65°C. The tailing buffer was made by mixing 400 µL of 5× TdT buffer (500 mM potassium cacodylate pH 7.2, 10 mM CoCl_2_ and 1 mM DTT) (Invitrogen) with 15 µL 100 mM dATP and 585 µL RNase free water.

Next, we added 90 µL of PCR mix to the resulting products and performed the following PCR program: 25 cycles of 2 min denaturation at 94°C, 2 min annealing at 42°C and 6 min (extending 10 sec each cycle) extension at 72°C. This was followed by, after adding extra 1 µL (5 U/µL) AmpliTaq (Roche), the same PCR program but without prolonging the extension time each cycle. For each PCR reaction, we mixed 53.5 µL water, 10 µL 10× PCR buffer II (100 mM Tris-HCl pH 8.3 and 500 mM KCl), 10 µL 25 mM MgCl_2_, 9 µL 200 µM AL-1 primer, 4 µL 100 mM dNTP, 1 µL 5% v/v Triton X‑100, 0.5 µL 20 mg/mL bovine serum albumin and 2 µL AmpliTaq (5 U/µL). After the PCR, we ran 5 µL of each amplified product in a 1.5% agarose gel. If the amplification is successful, you should see a smear extending from around 300 bp to 1200 bp (around 1 μg of DNA). However, frequently you can also see a smear in your negative (no cell) controls. This has been described previously (1) and is thought to result from bacterial contaminants present in the solutions. In order to identify successfully amplified cells, we prepared a Southern blot and probed it for a housekeeping gene (GAPDH). See Supplementary Protocol 1 for a comment on the GSC RT-PCR method.

The Global Single Cell Reverse Transcription-Polymerase Chain Reaction (GSC RT-PCR), originally described by N. Iscove and collaborators *(1,* 2), is a method that amplifies the messenger RNA contained in one cell. It consists of a limited RT reaction performed immediately after a cell is lysed, followed by a polyadenylation step, and PCR amplification of the cDNA obtained in the previous reactions. The cDNA molecules generated (poly-A cDNA) possess a 5’ oligo-dT tract and a 3’ oligo-dA tail, flanking 300 to 1500 bp of the 3’ end of the genes amplified. The combination of two different reverse transcriptases minimizes any sequence bias that each enzyme may possess individually and the 15 minute limit imposed on the RT reaction limits the average sizes of the reverse transcripts to less than approximately 1500 bp. The buffer used for tailing the reverse transcripts contains 100-fold more dATP than the initial solution, which ensures that the deoxynucleotide incorporated at the 3’ end of the reverse transcripts is almost exclusively dATP. The fact that the original reverse transcripts are limited in size minimizes amplification bias due to differences in size: smaller molecules are more likely to be amplified successfully than larger molecules during each round of PCR. The drawback is that only the most 3’ sequences from each gene become amplified, i.e., the poly-A cDNA pool is 3’ biased. However, since most gene libraries (including ESTs) are themselves 3’ biased, this should not interfere with our ability to detect the presence of a specific gene. There is inevitably some degree of variation introduced by our experimental procedures, but this issue has been addressed by the authors of the method who have found that the total variation induced by the method was in the range of 5 fold (3).

As previously described (1), we have found that both the concentration of magnesium and primer are critical for the success of the amplification. Minor deviations from the ones described decrease the reaction yield to the point of no detectable amplification. The AL-1 primer (ATT GGA TCC AGG CCG CTC TGG ACA AAA TAT GAA TTC T_24_) has been described previously and contains a EcoRI restriction site flanked by non-specific sequences, followed by an oligo-dT sequence that primes the poly-A tracts in the cDNAs. We have tried other primers that also work and may be useful to introduce T7 promotor sequences or other restriction sites. Each primer has its own optimal concentration.

### Target fragmentation

The remaining poly-A cDNA was purified in a PCR cleanup column (Qiagen, Valencia, CA, USA) and eluted in 50 µL 10 mM Tris-HCl pH 8.5. We then used a Speed Vac machine (Jouan, Winchester, VA, USA) or Microcon-30 filter devices (Millipore, Billerica, MA, USA) to concentrate 25 µg of poly-A cDNA to 67.5 µL and added 14.2 µL of buffer mix. Buffer mix was prepared by combining 10.5 µL One‑Phor‑All™ (Amersham, Piscataway, NJ, USA) buffer (100 mM Tris-acetate pH 7.5, 100 mM magnesium acetate and 500 mM potassium acetate) to 6.6 µL 25 mM CoCl_2_. We next added 0.5 U of DNase I (1 U/µL) (Invitrogen) and 9.5 µL of its respective 1× buffer (20 mM Tris‑HCl pH 8.4, 2 mM MgCl_2_, 50 mM KCl) and incubated the reaction for 3 min at 37°C, followed immediately by placement in a boiling water bath for 15 min*(4,* 5). Adequate fragmentation of the cDNA should generate segments averaging 50 bp. The optimal concentration of DNase I and digestion time have been titrated for our reaction, by running a digested sample aliquot on a denaturing 10% PAGE together with an appropriate molecular weight marker.

Because the material generated in this process is dsDNA and not biotinylated aRNA, we had to adapt the conventional Affymetrix protocol by end-labeling the cDNA with biotinylated ddATP *(4,* 5), after determining the optimal conditions for its random digestion with DNase I into fragments averaging 50 bp. The optimal concentration of DNase I and digestion time have been titrated for our reaction, by running a digested sample aliquot on a denaturing 10% PAGE together with an appropriate molecular weight marker. The targets thus produced were then incubated with Murine Genome U74A version 2 microarrays according to the standard protocol (Affymetrix). Since the probes present on these arrays correspond mostly to the 3’ ends of the genes represented, the fact that these targets are 3’ biased should not affect the results significantly, provided that an appropriate method of analysis is chosen.

### Target labeling

In order to end-label the cDNA fragments with biotin, we next added 3.12 µL of 1 mM N^6^‑biotinylated ddATP (PerkinElmer, Boston, MA, USA) and 4.25 µL of TdT (15 U/µL) (Invitrogen) and incubated the reaction at 37°C for 2 hours*(4,* 5).

### Microarray incubation

The biotin-labeled fragments were used directly as targets for GeneChip™ Murine Genome U74A version 2 microarrays (Affymetrix, Santa Clara, CA, USA) according to the Affymetrix standard protocol, but scaling down reactions to a final hybridization mixture volume of 250 µL. After hybridization with 200 µL of this solution (equivalent to 20 µg of cDNA), the arrays were incubated according to the Affymetrix protocol (antibody amplified) with phycoerythrin-conjugated streptavidin.

### Microarray analysis

The raw intensity data for each probe was collected with Microarray Suite version 5.0 software, MAS5, from Affymetrix (http://www.affymetrix.com). Expression levels for each of the genes represented in the array was computed by the Bioconductor implementation of the RMA (Robust Multichip Analysis) method (http://www.bioconductor.org). The primary data used in our work is compiled in Supplementary Table S5. Groups of samples were compared using BRB-ArrayTools package (http://linus.nci.nih.gov/BRB-ArrayTools.html) for Excel software (Microsoft, Redmond, WA, USA). A gene was considered to be differentially expressed between groups if a Student’s t-test statistic had an associated probability (P) less than 0.05. The use of the t statistic is more adequate for our type of experiments than the application of an absolute threshold for fold change. On the one hand, genes that have small fold changes but consistent levels (i.e., low variance) within different groups of samples, will be tagged as differentially expressed. On the other hand, genes that display high variability, either reflecting true biological fluctuations or limitations of the amplification method, will not be considered to be different between groups because their variances will be too high. Consequently, several false discoveries of genetic differences between groups will be avoided.

### Quantitative real time PCR (Q-PCR) validation for population expression levels

We designed primers for a total of 22 randomly selected genes: 7 whose expression levels were significantly higher in T cells, 8 in SP cells; and 7 not statistically different (Supplementary Table S2). We isolated total RNA from a minimum of 200,000 SP or CD8 cells, pooled from different sorting experiments, using the RNeasy kit (Ambion, Austin, TX, USA). The RNA was digested with DNAse I (Invitrogen) and resuspended in RNase free water (Promega) at a final concentration corresponding to 20,000 cells per microliter. Each 10 µL of RNA solution was incubated with 1 µL of 500 µg/mL oligo-dT_12-18_ (Invitrogen) and 1 µL of 10 mM dNTP (Invitrogen) at 65°C for 5 min. We then added 4 µL of first strand buffer (Invitrogen), 2 µL of 1 M DTT (Invitrogen) and 1 µL of RNAse out (Invitrogen) and kept the mixture at 42°C for 2 min. Finally, we performed a reverse transcription reaction by adding 1 µL of SuperScript (Invitrogen). No RT controls were done in parallel for each initial RNA sample and used to assess contamination with undigested DNA. The cDNA obtained had a final concentration corresponding to 10,000 cells per microliter.

Each Q-PCR reaction was performed by mixing 5 µL of 10× PCR buffer with SYBR Green (PerkinElmer), 6 µL of 25 mM MgCl_2_, 4 µL of 12.5 mM dNTP, 0.25 µL AmpliTaq (5 U/µL) (PerkinElmer), 3 µL of 100 mM forward primer, 3 µL of 100 mM reverse primer, 0.25 µL of cDNA template (equivalent to approximately 2500 cells) and 28.5 µL of RNAse free water (final reaction volume 50 µL).

The PCR program used was the following: 2 min warm-up at 50°C; 10 min denaturation at 95°C; 45 cycles of 1 min annealing and extension at 60°C and 15 sec denaturation at 95°C; 20 sec annealing at 60°C and 20 min ramp up to 95°C (for melting curve acquisition). The reactions were performed in an ABI 7900HT (Applied Biosystems, Foster City, CA).

No-template controls were always performed for each primer pair and these PCR reactions were consistently negative for the presence of amplicons. All reactions were run in duplicate and the mean threshold cycle (C_T_) was calculated for each pair of reactions performed for each of the two populations (C_T_^SP^ and C_T_^CD8^). These were normalized for beta-actin levels in each RNA sample (C'_T_^SP^ = C_T_^SP^ – A_T_^SP^ and C'_T_^CD8^ = C_T_^CD8^ – A_T_^CD8^, where A_T_ is threshold cycle for beta-actin in a particular sample). The log fold change for each gene is given by LFC_QPCR_ = C'_T_^SP^ – C'_T_^CD8^. Every selected gene was tested in two independent RNA samples obtained from each population. An average log fold change (Log QPCR) between the two populations was obtained for each of the 22 genes studied.

A parallel value was calculated from the RMA data obtained from 10-cell microarray experiments. The average expression levels for 6 SP samples (performed in duplicate) and 3 CD8 samples (idem) were calculated (E'^SP^ and E'^CD8^). Since RMA expression levels are in a logarithmic scale, the log fold change is given by LFC_RMA_ = E'^SP^ – E'^CD8^ (Log RMA). Corresponding log fold changes (Log QPCR and Log RMA) for each gene were plotted in the same graph. A linear regression model was fitted to the values obtained.

### Q-PCR validation for single cell experiments

We sorted single HSC into single wells of a 96-well plate containing 4 μl of the lysis buffer used in our GSC RT-PCR reactions. We included both negative and positive controls on each plate: wells containing only lysis buffer (no cell) were used as negative controls, and wells containing lysis buffer plus 25 stem cells were used as positive controls. A single gene was analyzed per 96-well plate to minimize cross-contamination. Each 96-well plate was heated at 65°C for 1 min and 25°C for 2min. We performed reverse transcription reactions by adding 0.5μl of Superscript II reverse transcriptase (Invitrogen) followed by incubation of the plate at 42°C for 60 min.

Following reverse transcription, cells were assayed for gene expression using multiplexed Q-PCR, which simultaneously detected 18S rRNA and our gene-of-interest. Each reaction was performed by mixing 25 μl TaqMan® universal PCR master mix (Applied Biosystems), 2.5 μl 18S rRNA endogenous control (VIC/MGB), 2.5 μl gene-of-interest TaqMan® gene expression assay (FAM/MGB), and 16 μl nuclease-free water. The TaqMan® gene expression assays (Applied Biosystems) used included Mm00484032_g1 (Ctla2a), Mm00493153_m1 (Lmo2), Mm00493153_m1 (Lyl1), Mm00441665_m1 (Tal1) and Mm00445212_m1 (c-Kit). We utilized the following cycling parameters: stage 1, 50°C for 2 min; stage 2, 95°C for 10 min; and stage 3, 94°C for 15 sec and 60°C for 1 min. Stage 3 was repeated for a total of 60 cycles. A single cell Q-PCR was considered successful when the C_T_ for 18S was within the range of 26 to 30; the average 18S signal for all tested single cells was 28.5, while the average 18S CT for negative control wells was 34. A gene was considered to be expressed when it had a detectable signal within 60 cycles of amplification.

### References

1. G. Brady, N. N. Iscove, *Methods Enzymol* **225**, 611-23 (1993).

2. G. Brady, M. Barbara, N. Iscove, *Methods in Molecular and Cellular Biology* **2**, 17-25 (1990).

3. L. H. Brail *et al.*, *Mutat Res* **406**, 45-54. (1999).

4. R. J. Cho *et al.*, *Mol Cell* **2**, 65-73. (1998).

5. R. J. Cho *et al.*, *Proc Natl Acad Sci U S A* **95**, 3752-7. (1998).
